# Natural noncoding *pumilio* variants retune value-coding interneurons to bias *Drosophila* oviposition decisions

**DOI:** 10.1126/sciadv.aed1338

**Published:** 2026-05-08

**Authors:** Dorsa Motevalli, Robert Alfredson, Sydney Fogleman, Emmanuel Medrano, Yang Chen, William I. Silander, Toshihide Hige, Ulrich Stern, Chung-Hui Yang

**Affiliations:** ^1^ Dept. of Neurobiology, Duke University Medical School, Durham, NC 27710, USA.; ^2^ Dept. of Biology, University of North Carolina, Chapel Hill, NC 27599, USA.; ^3^ Dept. of Cell Biology and Physiology, University of North Carolina, Chapel Hill, NC 27599, USA.; ^4^ Integrative Program for Biological and Genome Science, University of North Carolina, Chapel Hill, NC 27599, USA.

## Abstract

How natural regulatory genetic variation creates innate biases in economic decisions through modifying circuit structure and function is rarely explored. Here, we trace this link in a simple value-based decision in *Drosophila*: where to oviposit. Although laboratory flies (*w^1118^*) reject sucrose for a plain option, a wild-caught African strain accepts sucrose. This decision difference maps to three African-specific intronic SNPs in *pumilio* (*pum*), an RNA binding translational repressor. These SNPs down-regulate *pum*, derepressing its target, the sodium channel *paralytic* (*para*), in a pair of GABAergic interneurons that encode option value. Elevated *para* boosts neuronal excitability, compresses value contrast between sucrose and plain options, and promotes sucrose acceptance. Selectively reducing *pum* or overexpressing *para* in these neurons converts the laboratory flies’ physiology and behavior to the African phenotype. These findings offer a genome-to-circuit-to-behavior framework, revealing how subtle regulatory polymorphisms fine-tune circuit properties to diversify environment-appropriate decision biases in nature.

## Teaser

Natural genetic variation fine-tunes circuit properties to shift value-based egg-laying decisions in fruit flies.

## INTRODUCTION

Natural genetic variation offers a powerful window into how genes fine-tune neural circuits to produce behavioral diversity. Single-gene variants, for example, modulate pair-bonding in voles, feeding behavior in worms, sugar-bait avoidance in cockroaches, and, as shown recently, photoperiod adaptation in *Drosophila* (*1*–*4*). Yet, we know little about how genetic variation adjusts the neural circuits that underlie value-based economic decisions—the process of ranking alternatives by their subjective worth. Ventral pallidum, orbitofrontal cortex, and dopaminergic (DA) neurons have been implicated in assigning subjective option value in mammals (*5*–*8*), but how genetically encoded circuit differences give rise to innate decision biases remains largely unexplored.

**Fig. 1.**
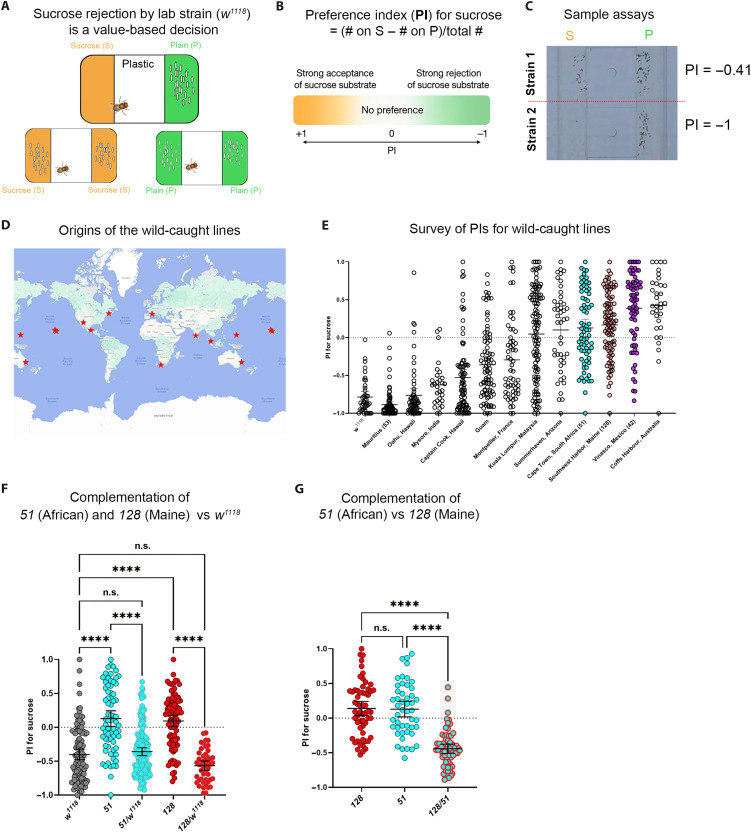
Genetic control of natural variation in sucrose preference during oviposition. (**A**) Value-based egg-laying choice between sucrose (150 mM sucrose in 1% agarose) and plain (1% agarose). The lab strain *w^1118^* accepts either substrate alone but rejects sucrose in a sucrose versus plain choice. (**B**) Definition of the sucrose PI = (eggs on sucrose − eggs on plain)/total eggs. (**C**) Representative oviposition choices from individual females (see fig. S1 for automated counting). (**D**) Geographic origins of wild-caught lines tested. (**E**) PI distributions across strains; each point represents one fly (*n* = 42, 85, 62, 30, 87, 85, 117, 83, 45, 70, 90, 71, and 33). (**F**) PIs of parental strains (*w^1118^*, *51*, and *128*) and their F__1__ progeny (*w^1118^/51* and *w^1118^/128*) (*n* = 111, 70, 88, 149, and 48). (**G**) PIs of parental strains *51* and *128* and their F__1__ (*128/51*) (*n* = 58, 48, and 64). Homozygous lines are at times denoted *51/51* or *128/128* (as opposed to *51* and *128*) where emphasis is needed. Statistical tests were performed using one-way ANOVA with Tukey’s multiple comparisons test. We indicated *P* value ranges using n.s.: *P* ≥ 0.05 and ****P < 0.0001. All error bars represent 95% CIs.

*Drosophila* egg-laying site selection has emerged as a powerful model for studying value-based decisions (*9*–*13*). Female flies search, evaluate, and compare potential sites before depositing each egg (*10*, *12*). They are capable of “choosing the greater of two goods,” a hallmark of value-based decisions. Standard laboratory strains such as *w^1118^* readily accept either a sucrose substrate or a plain substrate when only one type is offered, but they reject sucrose if both are available (Fig. 1A) (*10*–*12*, *14*). A recent study has identified the egg-laying command neurons—oviposition descending neurons (oviDN)—in the brain (*14*). oviDN activity exhibits a “ramp-to-threshold” pattern (*13*) as females deliberate, reminiscent of the “drift-to-bound” dynamics of decision-making neurons in higher organisms (*5*, *6*). These properties, together with the extensive genetic and circuit manipulation toolkit and the recently completed connectome of an adult female brain (*15*–*17*), make *Drosophila* egg-laying decisions ideal for exploring how genetically encoded circuit variation drives variation in decision outcomes in nature.

Here, we exploit a notable difference in sugar preference during egg laying between the standard laboratory strain *w^1118^* and a wild-caught strain from Africa to investigate how natural genetic variation sculpts circuitry to generate distinct innate decision biases. By combining behavior analysis, genome-wide SNP mapping, and connectome-guided circuit interrogation, we identify differential expression of *pumilio* (*pum*), encoding an RNA binding protein (*18*, *19*), as a key determinant governing the two strains’ distinct choices between sucrose and plain substrates. Specifically, strain-specific SNPs tune *pum* expression, which, in turn, modulates the level of the voltage-gated sodium channel encoded by *paralytic* (*para*), a known target of *pum* (*20*). This *pum*-adjusted difference in excitability alters the ratio of sucrose-evoked versus plain-evoked responses in a pair of value-coding—and decision-critical—inhibitory interneurons we identified, biasing the animal’s behavioral choice. Together, these results demonstrate that genetically encoded differences in *pum* expression can modify circuit properties, offering a rare end-to-end demonstration of how natural variation in a gene-regulatory region reshapes computations within a defined microcircuit to yield strain-specific decision outcomes that may better suit each habitat.

## RESULTS

### Sucrose preference varies among wild-caught strains and is under simple genetic control

To investigate whether strains originating from diverse locations in the world display distinct innate oviposition decision biases, we surveyed a collection of wild-caught lines from the Cornell Stock Center for their sucrose (150 mM)–versus–plain choices (Fig. 1, A to E). We used custom apparatuses and egg-counting code to assay preferences of many animals in parallel (*21*) (fig. S1) and calculated each individual’s sucrose preference index (PI) as a quantitative measure of decision bias (Fig. 1, B and C). As anticipated, not all wild-caught strains rejected sucrose: Although our standard laboratory strain (*w^1118^*) rejected sucrose and had a mean PI of ~−0.7 in this survey, many of the wild-caught strains were either indifferent to sucrose or even preferred sucrose for egg laying (Fig. 1E).

To probe the genetic complexity underlying sucrose acceptance differences, we performed complementation to test how F_1_ progeny from crosses of *w^1118^* to either the African strain (line 51) or the Maine strain (line 128) (both sucrose-accepting) would choose. F_1_ progeny from both crosses (*w^1118^/51* and *w^1118^/128*) behaved more like *w^1118^* and rejected sucrose (Fig. 1F). Notably, F_1_ progeny (*128/51*) of the two sucrose-accepting parental strains rejected sucrose (Fig. 1G). These results suggest that not only sucrose acceptance is a recessive trait but also the causal variants in nature occur in at least two complementation groups; that is, they have at least two distinct genetic origins. [In addition, because the *128/51* F_1_ progeny are *white^+^* (*w^+^*) and rejected sucrose, sucrose rejection exhibited by *w^1118^*, which carries a loss-of-function mutation in *w*, and its F_1_ progeny with lines 51 and 128 is unlikely to be caused by mutation in *w*, a gene encoding an ABCG-type transporter known to affect certain behaviors (*22*, *23*).] Together, these observations suggest that (i) the genetic control of how animals value sucrose in this decision may be relatively simple and (ii) the SNPs that drive sucrose rejection versus acceptance could, in principle, be mapped. Here, we use the term SNPs in a broad sense to include both single-nucleotide polymorphisms and indels (insertions and deletions).

### Intronic SNPs in the RNA binding translational repressor *pumilio* (*pum*) in the African strain drive sucrose acceptance

**Fig. 2.**
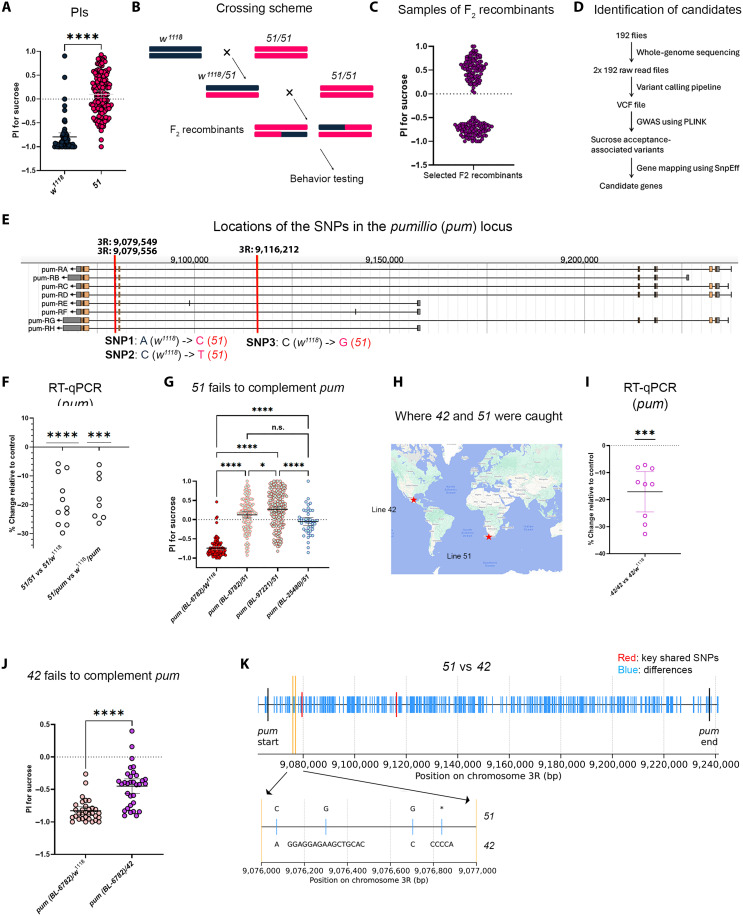
Intronic *pum* SNPs in wild-caught strains reduce *pum* and increase sucrose acceptance. (**A**) PIs of *w^1118^* and line *51* (*n* = 63 and 141). (**B**) Crossing scheme used to generate F_2_ recombinants. (**C**) PIs of representative F_2_ recombinants. (**D**) GWAS pipeline. (**E**) *pum* gene structure with three significant intronic SNPs highlighted. (**F**) Brain RT-qPCR showing *pum* expression in *51/51*, *51/w^1118^*, *51/pum*, and w^1118^/pum (*n* = 11 and 8; biological replicates), normalized to *actin5C*. (**G**) Complementation analysis of *pum* alleles with *w*^1118^ and *51* (*n* = 111, 112, 182, and 44). (**H**) Geographic origins of lines 42 (Mexico) and 51 (Africa). (**I**) Head RT-qPCR showing *pum* expression in *42/42* and *42/w^1118^* (*n* = 9). (**J**) Complementation test between a *pum* mutation and line 42 (*n* = 32 and 32). (**K**) Sequence divergence between lines *51* and *42*; the bottom panel shows an expanded view of the bracketed region. Statistical tests were performed using unpaired two-sided Welch’s *t* tests [(A) and (J)], one-sample two-sided *t* tests [(F) and (I)], and one-way ANOVA with Tukey’s multiple comparisons test (G). We indicated *P* value ranges using n.s.: *P* ≥ 0.05, *P < 0.05, ***P < 0.001, and ****P < 0.0001. All error bars represent 95% CIs.

We next attempted to map the SNPs—and thus the genes—underlying the laboratory (*w^1118^*) and African (line 51) strains’ divergent valuation of sucrose (Fig. 2A). We chose line 51 in part because some other sucrose-accepting lines (e.g., line 42) appeared genetically more complex and may harbor a union of the distinct variants underlying sucrose acceptance in lines 51 and 128 (fig. S2). We generated animals with hybrid genomes by backcrossing the F_1_ female progeny of the *w^1118^* × line 51 cross to line 51 males, so that each F_2_ female carried one nonrecombinant chromosome from line 51 and one recombinant chromosome (of line 51 and *w^1118^*) (Fig. 2B). We then assayed the PI for sucrose of many such F_2_ individuals (Fig. 2C) and selected 192 of them for full-genome sequencing. We next conducted a genome-wide association study (GWAS) with PLINK analysis (*24*) to identify SNPs associated with PI (Fig. 2D). Three significant, high-impact (i.e., large effect) SNPs are particularly notable because they lie within the introns of the same gene: *pumilio* (*pum*) (Fig. 2E and tables S1 and S3), which encodes an RNA binding translational repressor (*18*, *19*).

Given that sucrose acceptance in the egg-laying decision is recessive, we speculated that the SNPs in line 51 reduce *pum* expression. This is because recessive alleles often reflect reduced gene function. *pum* expression was lower in the brains of line 51 flies than controls (Fig. 2F). We next asked whether line 51 would fail to complement *pum* by assessing the F_1_ progeny of line 51 and *pum* mutants, a direct test of whether reduced *pum* drives sucrose acceptance. Although F_1_ progeny from control crosses (*pum/w^1118^* and *51/w^1118^*) rejected sucrose, *pum/51* accepted sucrose; this was true for three different *pum* mutant lines from distinct genetic backgrounds (Figs. 1F and 2G). We also asked whether the SNPs we identified in line 51 might be present in other sucrose-accepting wild-caught strains. By looking for other lines that failed to complement line 51 (fig. S2), we found that line 42—collected in Mexico—harbored the same SNPs (Fig. 2H and table S2). Consistent with the idea that these SNPs reduce *pum* expression, line 42 flies showed lower *pum* expression (Fig. 2I) and failed to complement a *pum* mutation (Fig. 2J). Notably, despite sharing the three key intronic SNPs in *pum*, these two strains are otherwise genetically divergent. Even within the *pum* locus, they differ at many positions, including several large insertion-deletion variants, consistent with their distinct geographical origins (Fig. 2K and table S2).

Together, our analyses suggest that the level of *pum* expression—regulated in part by intronic variation in *pum*—is crucial for how *Drosophila* value sucrose relative to plain substrates during egg-laying decisions. Specifically, higher *pum* expression in the laboratory strain *w^1118^* drives sucrose rejection, whereas lower *pum* levels in the African strain line 51 (and the Mexican strain line 42) promote sucrose acceptance.

### Sucrose rejection in the lab strain (*w^1118^*) is mediated by a pair of interneurons Earmuff

**Fig. 3.**
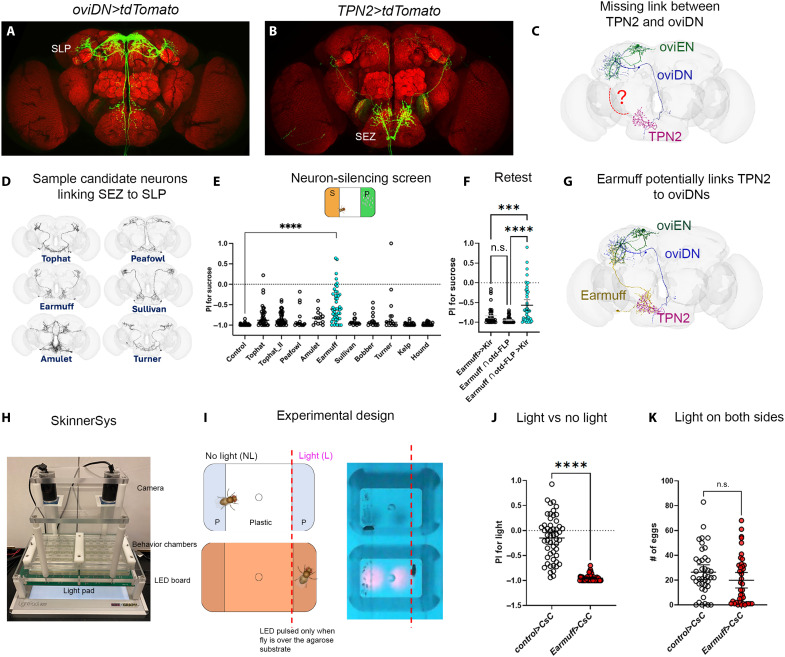
Earmuff neurons are necessary and sufficient for sucrose rejection in the lab strain (*w^1118^*). (**A** and **B**) Confocal images of oviDN neurons and TPN2 (see fig. S3). (**C**) FlyWire reconstructions of TPN2, oviEN, and oviDN in one hemisphere; dashed line indicates missing component(s) in the TPN2 → oviEN/oviDN pathway. (**D**) Example SEZ → SLP projection neurons included in the functional screen. (**E**) PIs following Kir-mediated silencing of candidate neurons (*n* = 36, 37, 45, 15, 15, 49, 16, 16, 16, 24, and 24). (**F**) Independent validation of Earmuff silencing (*n* = 43, 47, and 45). (**G**) FlyWire reconstructions of TPN2, Earmuff, oviEN, and oviDN. (**H**) SkinnerSys rig enabling real-time optogenetic stimulation of 40 flies in parallel. (**I**) Stimulation paradigm for the light/no-light choice assay. (**J**) PIs of *Earmuff>CsChrimson* flies and controls in the light/no-light choice assay (*n* = 52 and 54). (**K**) Total eggs laid when light is triggered on both substrates (*n* = 41 and 40). Statistical tests were performed using unpaired two-sided Welch’s *t* tests [(E), (J), and (K)] and one-way ANOVA with Tukey’s multiple comparisons test (F). All transgenic lines were generated in the *w^1118^* background. We indicated *P* value ranges using n.s.: *P* ≥ 0.05 and ****P < 0.0001. All error bars represent 95% CIs.

**Fig. 4.**
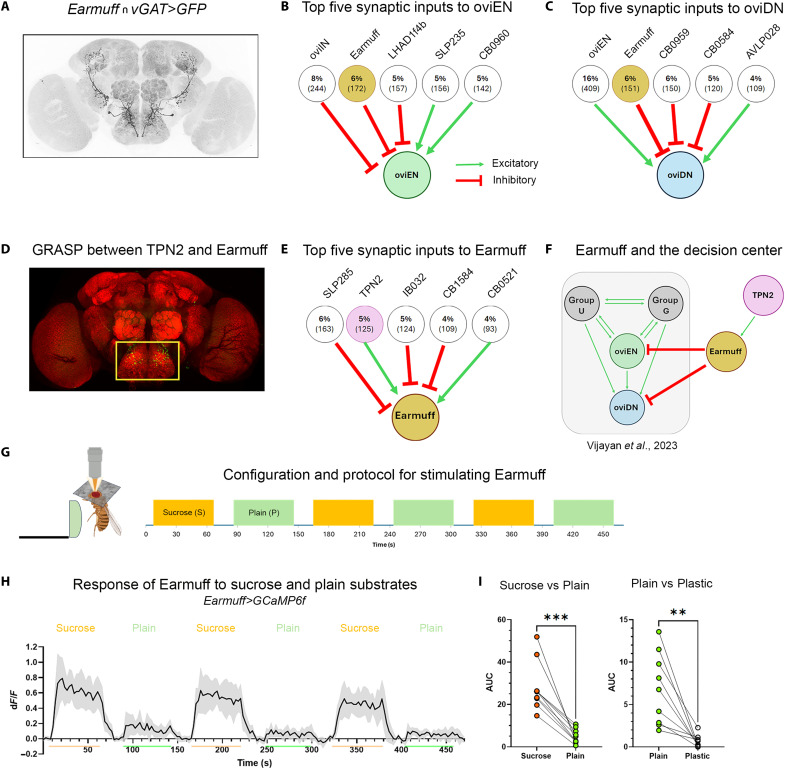
Earmuff neurons preferentially respond to sucrose in *w^1118^* and inhibit the decision node. (**A**) *vGAT-LexA* expression identifies Earmuff neurons as GABAergic. (**B** and **C**) Top presynaptic partners of oviEN and oviDN from FlyWire. (**D**) GRASP signal revealing TPN2 → Earmuff contacts in the SEZ. (**E**) Top presynaptic partners of Earmuff from FlyWire. (**F**) Circuit model adapted from Vijayan *et al.* (*13*), published under CC BY 4.0 (https://creativecommons.org/licenses/by/4.0/), with Earmuff added as an input to oviEN/DN. (**G**) Ca^2+^ imaging configuration and protocol. (**H**) Mean Earmuff Δ*F*/*F* responses to sucrose and plain substrates (*n* = 10; shaded, 95% CI). (**I**) Area under the curve (AUC) comparisons for sucrose versus plain (*n* = 10) and plain versus plastic (*n* = 9). Statistical tests were performed using paired two-sided *t* tests. AUCs were used because Earmuff responses were sustained. We indicated *P* value ranges using *P < 0.05, **P < 0.01, ***P < 0.001. All error bars represent 95% CIs.

To pinpoint the neural substrates through which reduced *pum* expression promotes sucrose acceptance in the African strain line 51, we next identified—in *w^1118^*—the circuit that converts the sensory qualities of egg-laying options into value signals relayed to the command neurons oviDN (Fig. 3A). A recent study showed that leg sweet taste neurons and their postsynaptic targets, TPN2 neurons (Fig. 3B), are necessary and sufficient to devalue sucrose as females deliberate between sucrose and plain substrates (*21*) (fig. S3). In addition, a few recurrently connected excitatory neurons (e.g., oviEN) that signal to oviDN have been reported (*13*, *14*). However, circuit components that bridge the TPN2 axons, which terminate in the subesophageal zone (SEZ) (fig. S3), and the recurrently connected groups presynaptic to oviDN, which arborize in the superior lateral protocerebrum (SLP), remained unknown (Fig. 3C). We performed a small-scale screen in which we silenced neurons projecting from the SEZ to the SLP with the Kir channel, using the morphological criteria and *split-GAL4* lines described by Sterne *et al.* (*25*) (Fig. 3D). Inactivating neurons labeled by one specific line consistently increased sucrose acceptance (Fig. 3, E and F). These neurons are called Earmuff, likely because their morphology resembles a pair of earmuffs (Figs. 3G and 4A). (Note that all the transgenic tools we used in this work were generated in the genetic background of the standard lab strain, which rejects sucrose when flies are assayed individually.)

Having found that Earmuff neurons are necessary for proper sucrose rejection, we asked whether their artificial activation is sufficient to promote rejection. We expressed the channelrhodopsin *CsChrimson* (*26*) in these neurons and assessed how flies chose between two plain substrates with light pulsed while flies were over one of the substrates (Fig. 3, H to K). We implemented this using SkinnerSys, a system we developed that can track 40 animals in parallel (per apparatus) and deliver light in closed loop (*21*) (Fig. 3, H and I). *Earmuff>CsChrimson* (*CsC*) flies robustly rejected the illuminated plain option, whereas control flies were indifferent between the two options (Fig. 3J). To determine whether optogenetic activation of Earmuff neurons simply suppresses egg laying or instead “devalues” the option on which it occurs, we assessed behavior when both plain options were illuminated (Fig. 3K). Flies did not significantly reduce their egg-laying rate when their Earmuff neurons were optogenetically activated on both options (Fig. 3K), suggesting that Earmuff activation reduces the value of an option rather than abolishing egg laying. This mirrors how flies behave toward sucrose: They refrain from egg laying on sucrose when a plain substrate is available but accept sucrose for egg laying when it is the only option (Fig. 1A).

### Earmuff neurons inhibit the decision-maker and encode option values

We next investigated the circuit mechanism by which Earmuff neurons promote sucrose rejection in *w^1118^*. Assessing the relationship between Earmuff and the command neurons oviDN in the FlyWire connectome revealed two noteworthy features. First, Earmuff neurons are annotated as GABAergic, and we confirmed this by showing that the promoter for *vGAT*, a marker for GABAergic neurons, was active in them, as was the gene product (Fig. 4A and fig. S4E). Second, Earmuff neurons are annotated to provide direct synaptic input both to oviEN—the key excitatory input to oviDN (*13*, *14*)—and to oviDN themselves (Fig. 4, B and C). Earmuff neurons are annotated to provide the second-strongest inhibitory input to each target (Fig. 4, B and C). Consistent with these connectivity predictions, we found that stimulus-evoked responses in oviENs increased when synaptic output of Earmuff neurons was blocked by tetanus toxin (TNT) (fig. S4D). These results, together with the behavioral phenotypes, suggest Earmuff neurons contribute to sucrose rejection by inhibiting the decision-maker (oviDN) and its strongest excitatory input (oviEN).

How could inhibition from Earmuff neurons enable flies to reject sucrose while accepting plain substrate? One possibility is that Earmuff neurons are activated specifically by sweet taste, so that only the sucrose option triggers their inhibition of the decision-making circuit. Ca^2+^ imaging showed that Earmuff neurons responded strongly to sucrose solution, “opto sugar,” and sucrose substrate (fig. S4, A and B) and were functionally connected to sweet taste projection neuron TPN2 previously shown to be important for sugar rejection (*21*) (Fig. 4, D and E, and fig. S4C). However, we found that Earmuff neurons responded to the plain agarose substrate too, albeit consistently at a reduced level (Fig. 4, G to I). However, they showed little or no response to a hard plastic surface (Fig. 4I). These results suggest that Earmuff neurons are sensitive to both sweet taste and texture of an egg-laying substrate, in keeping with a recent connectome-based analysis showing that multimodal integration is a common theme of higher-order “taste neurons” (*27*).

Our combined behavioral, anatomical, and physiological evidence thus indicates that Earmuff neurons encode innately specified, substrate-specific “negative values” for sucrose and plain options, enabling the decision-maker to distinguish between them. In *w^1118^*, the sucrose substrate activates Earmuff neurons strongly, leading to greater inhibition of the decision-maker and a lowered perceived value. Conversely, the plain substrate activates Earmuff neurons mildly, inhibiting the decision-maker less and therefore carrying a higher perceived value than sucrose. Curiously, although males lack oviEN/DN neurons, their Earmuff neurons are morphologically indistinguishable from those of females and are similarly connected to TPN2 neurons (fig. S5).

### *pum* expression level in Earmuff is important for strain-specific value coding and decision outcome

**Fig. 5.**
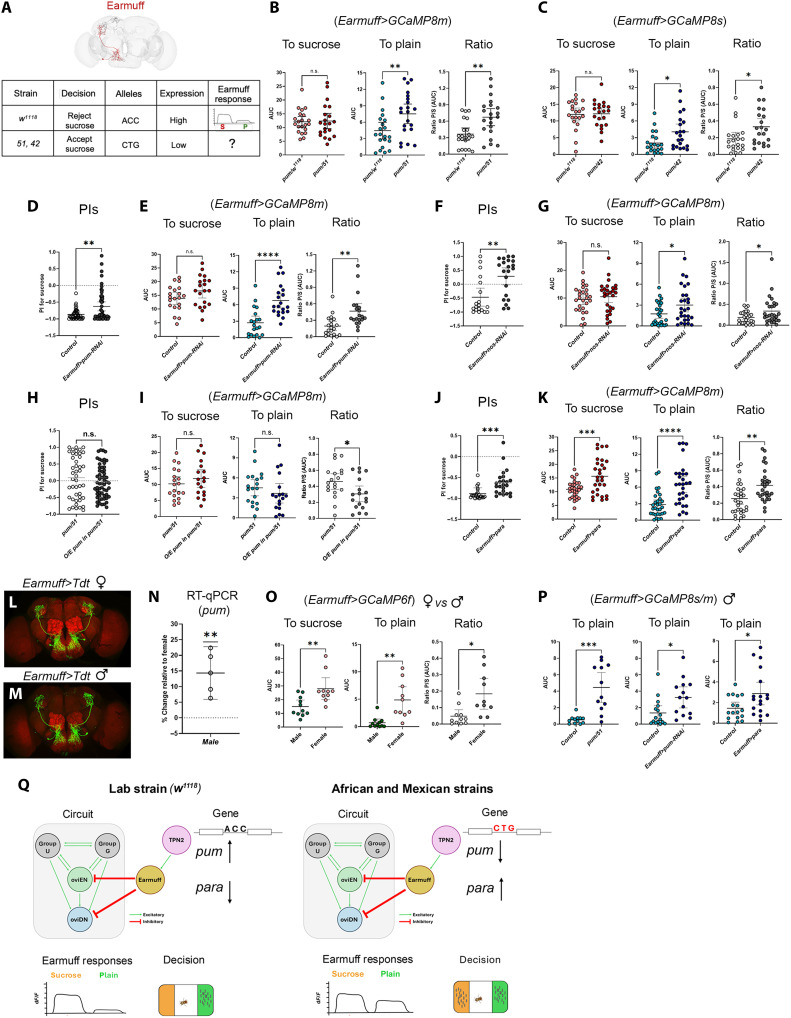
*pum* levels in Earmuff neurons set strain-specific sucrose preference. (**A**) Summary of behavioral and imaging results. (**B** and **C**) Earmuff responses in control versus *pum/51* or *pum/42* flies (*n* = 21 each), quantified as sucrose AUC, plain AUC, and plain/sucrose (P/S) ratio. (**D** and **E**) Earmuff-specific *pum* knockdown in *w^1118^*: PI (*n* = 44 and 50) and Earmuff responses (*n* = 21 and 28). (**F** and **G**) Earmuff-specific *nos* knockdown in *w^1118^*: PI (*n* = 19 and 21) and Earmuff responses (*n* = 27 and 28). (**H** and **I**) Earmuff-specific *pum* overexpression in *51/pum*: PI (*n* = 44 and 58) and Earmuff responses (*n* = 19 and 18). (**J** and **K**) Earmuff-specific *para* overexpression in *w^1118^*: PI (*n* = 22 and 24) and Earmuff responses (*n* = 30 and 29). (**L** and **M**) Comparison of female and male Earmuff neurons. (**N**) *pum* expression in female versus male brains (*n* = 5 biological replicates), normalized to *actin5C*. (**O**) Female versus male Earmuff responses in *w^1118^* (*n* = 10 and 11). (**P**) Male Earmuff responses in *pum/51* (*n* = 12 and 12), Earmuff-specific *pum* knockdown (*n* = 16 and 14), and Earmuff-specific *para* overexpression (*n* = 17 and 18). (**Q**) Model in which regulatory SNPs at the *pum* locus alter Earmuff excitability and sucrose-plain coding to bias sucrose preference. U, PAL02-DA neurons; G, PAL04-DA neurons plus unidentified cholinergic and possible peptidergic inputs. Statistical tests were performed using unpaired two-sided Welch’s *t* tests. We indicated *P* value ranges using n.s.: *P* ≥ 0.05, *P < 0.05, **P < 0.01, ***P < 0.001, and ****P < 0.0001. All error bars represent 95% CIs.

Having found that differential responses of Earmuff neurons to sucrose and plain substrates are likely critical for sucrose rejection in favor of plain in the lab strain *w^1118^*, we returned to ask whether sucrose acceptance in the African strain (line 51) may result from altered substrate responses of its Earmuff neurons (Fig. 5A). To assess how Earmuff neurons respond to substrates in the African strain, we used the trans-heterozygous *51/pum* flies as surrogates as they behaved similarly to homozygous *51/51* animals with respect to sucrose acceptance. We initially expected that Earmuff neurons in *51/pum* animals would have reduced responses to sucrose substrates. To our surprise, sucrose responses of Earmuff in these animals were comparable to controls, but their responses to plain increased significantly, elevating the response ratio of plain to sucrose (Fig. 5B and fig. S6). We also examined responses of Earmuff in trans-heterozygous *42/pum* flies (surrogates for the Mexican line) and found a similar phenotype (Fig. 5C and fig. S6). These results suggest that Earmuff’s ability to discriminate between sucrose and plain substrates is diminished in both sucrose-accepting wild-caught strains, lines 51 and 42.

To directly test whether the reduced discriminative capacity of Earmuff neurons plays a role in promoting sucrose acceptance in the wild-caught strains, we selectively manipulated *pum* expression in Earmuff neurons of the laboratory strain (*w^1118^*). We found that overexpressing RNA interference (RNAi) against either *pum* or *nanos* (*nos*), a known partner of *pum* (*28*, *29*), in Earmuff neurons led to increased sucrose acceptance (reduced sucrose rejection) in *w^1118^* (Fig. 5, D and F). In keeping with the behavioral phenotype, overexpressing these RNAi in Earmuff neurons increased the plain-to-sucrose response ratio (Fig. 5, E and G**,** and fig. S7). We also attempted to selectively increase *pum* in just the Earmuff neurons in the *51/pum* surrogate and found that, although behavior was not rescued, the plain-to-sucrose response ratio was rescued (Fig. 5, H and I). These results suggest that, although reduced *pum* in Earmuff neurons alters their substrate responses, thereby altering behavior, Earmuff neurons are not the only behaviorally relevant neurons affected by reduced *pum* in the African strain. The response ratio of TPN2, an important presynaptic partner of Earmuff, was altered as well (fig. S8).

How might altered *pum* expression in Earmuff neurons affect their response properties? Given that Earmuff neurons increased their plain response when *pum* was reduced, we were curious whether increased *paralytic* (*para*), encoding a voltage-gated sodium channel known to be repressed by the *Pum-Nos* complex (*20*, *29*), might play a role. We therefore overexpressed *para* specifically in Earmuff neurons of *w^1118^* and found that it increased both the plain-to-sucrose response ratio of Earmuff neurons and sucrose acceptance (Fig. 5, J and K, and fig. S7). Curiously, although overexpressing *pum-RNAi* reduced discrimination by selectively increasing Earmuff’s plain response, overexpressing *para* reduced discrimination by eliciting a larger increase in the plain response and a moderate increase in the sucrose response (Fig. 5, J and K, and fig. S7).

To further test the idea that *pum* down-regulation—and the consequent *para* elevation—enhances Earmuff neurons’ responses to the plain substrate, we examined these neurons in males of the laboratory strain (*w^1118^*) (Fig. 5, L and M). Because we found *pum* expression to be sexually dimorphic in *w^1118^*, with higher levels in male than in female brains (Fig. 5N), examining Earmuff neurons in *w^1118^* males allows us to assess substrate responses under naturally elevated *pum* expression. Male Earmuff neurons discriminated sucrose from plain substrates more clearly and had lower plain-to-sucrose response ratio (Fig. 5O). Plain responses in male Earmuff neurons increased when we selectively reduced *pum* or increased *para* expression in them or when assayed in the African strain surrogate background (Fig. 5P). Thus, our collective results support the notion that reducing *pum* in Earmuff alters their responses, likely by derepressing *para* and increasing excitability. In African and Mexican females, the resulting compressed sucrose-versus-plain response difference plays a role in driving their acceptance of sucrose for egg laying (Fig. 5Q).

## DISCUSSION

In this study, we establish a link between naturally occurring genetic variation and strain-specific innate decision bias in *Drosophila*. Our data suggest a path from intronic SNPs in the translational repressor *pum*, through value coding in a pair of decision-critical inhibitory neurons, to the resulting behavioral choice. We propose (Fig. 5Q) that variants in regulatory regions of *pum* in a wild-caught African strain—and in a Mexican strain—lower *pum* expression, thereby derepressing the voltage-gated sodium channel *para* in Earmuff neurons, which directly inhibit the decision center. The resulting increase in excitability compresses Earmuff’s value code for sucrose versus plain substrates, which is then inversely mirrored in oviDN. Furthermore, *pum* and *nos* knockdown or *para* overexpression specifically in Earmuff neurons in *w^1118^* recapitulated the wild-caught phenotype, whereas selectively overexpressing *pum* in Earmuff neurons in the surrogates shifted neural responses to more closely resemble those of *w^1118^*, demonstrating that tuning Earmuff activity can redirect choice. Together, these findings provide an end-to-end example of how natural regulatory variation reshapes circuit properties to alter behavior, and they point to a mechanism that may be broadly applicable given the evolutionary conservation of *pum* and *para*.

Are reduced *pum* expression—or elevated *para*—specifically in Earmuff neurons the sole cause of the altered sucrose preference in the two wild-caught strains? We think not. First, our PLINK association analysis identified several significant SNPs in other genes (tables S1 and S3), although their roles remain untested. Second, although we focused on Earmuff neurons here, additional decision-relevant nodes are likely also affected. For example, we observed similar effects of *pum* and *para* on TPN2 neurons, which are presynaptic to Earmuff (fig. S8). Furthermore, although reducing *pum* in Earmuff neurons of *w^1118^* altered both neural and behavioral responses, overexpressing *pum* in Earmuff neurons of the *51/pum* surrogates rescued neural, but not behavioral, responses. Possible explanations for the lack of behavioral rescue include incomplete restoration of *pum* to *w^1118^* expression levels or expression of an inappropriate isoform. However, it is also possible that neurons downstream of Earmuff are affected in the wild-caught strains (through reduced *pum* expression in those neurons), given how widely expressed *pum* is in the brain. Leading candidates include oviEN/oviDN and two groups of neurons reciprocally connected to oviEN (*13*) (Fig. 5Q): group G neurons (one pair of PAL04 dopaminergic neurons, one pair of cholinergic neurons, and one pair with unknown transmitter identity) and group U neurons (one pair of PAL02 dopaminergic neurons). Assessing the response properties of oviEN/oviDN in laboratory versus wild-caught backgrounds is a logical next step.

How could *pum* reduction selectively enhance the plain response in Earmuff neurons? One possibility is that Pum proteins are enriched at synapses receiving plain-signaling input relative to those receiving sucrose-signaling input. Thus, lowering *pum* could increase local sodium-channel abundance, selectively elevating responses to plain stimuli. Testing this hypothesis will require genetic access to the relevant presynaptic partners of Earmuff neurons and visualizing Pum-dependent translational repression at subcellular resolution. The sexually dimorphic Earmuff responses offer a tractable pilot: In males, Earmuff neurons’ plain response is markedly reduced compared with females, hinting at greater Pum enrichment at synapses receiving plain input. Alternatively, Earmuff responses to sucrose substrates may already be near maximal—a ceiling effect—such that increasing excitability would selectively amplify only the plain response.

Last, could down-regulation of *pum* be a general route to increased acceptance of sucrose over plain for oviposition in nature? We believe so. First, line 42 is not the only wild-caught strain that failed to complement line 51 (fig. S2), suggesting that reduced *pum* expression may recur across strains. Second, the key SNPs driving sucrose acceptance in line 128 (collected in Maine) are likely distinct from those we identified in line 51 because the two strains complemented each other (Fig. 1), yet *pum* expression in 128 was also reduced (fig. S2). This pattern suggests that SNPs outside the *pum* locus can nonetheless converge on reducing *pum* expression. Adjusting *pum* levels through diverse molecular mechanisms may therefore be a common evolutionary lever for retuning sucrose valuation for oviposition. What selection pressures might tip the lever? Possibilities include limited sugar availability, reduced predation risk on sugary substrates, and an altered sense of foraging cost (*30*).

## MATERIALS AND METHODS

### Fly husbandry

Flies were reared on molasses-based food and maintained in a 25°C incubator with 60% humidity. For most behavior and imaging experiments, we used mated females that were 6 to 14 days old. For experiments using RNAi to knock down gene expression, flies were aged up to 22 days to allow sufficient time for targeted mRNA to be depleted. See the table of abbreviated crosses for the flies used in each experiment. For a complete list of strains and the abbreviated genotypes used in this study, see Tables 1 and 2.

**Table 1.** Key resource table.

Reagent/resource	Source	Identifier
**Antibodies**		
NC82 (mouse)	DSHB	AB_2314866
Anti-RFP (rabbit)	Thermo Fisher Scientific	AB_560939
Donkey anti-mouse, Alexa Fluor 488	Thermo Fisher Scientific	AB_141607
Donkey anti-rabbit, Alexa Fluor 594	Thermo Fisher Scientific	AB_141637
Donkey anti-rabbit, Alexa Fluor 488	Thermo Fisher Scientific	AB_2535792
Donkey anti-mouse, Cy3	Jackson ImmunoResearch	AB_2340813
Anti-GFP (rabbit)	Thermo Fisher Scientific	AB_221569
Anti-V5 (mouse)	Thermo Fisher Scientific	AB_2556564
**Oligonucleotides**		
*pumilio*	Thermo Fisher Scientific	Dm02135527_m1
*actin5C*	Thermo Fisher Scientific	Dm02361909_s1
**Chemicals**		
Sucrose	Sigma-Aldrich	S0389-5KG
UltraPure Agarose	Invitrogen	16500500
All trans-Retinal	Sigma-Aldrich	R2500-100MG
UltraPure DNase/RNase-Free Distilled Water	Thermo Fisher Scientific	10977023
Active dry yeast	VWR	75860-348
Potassium Chloride	Sigma-Aldrich	P9541-1KG
SlowFade Diamond Antifade Mountant	Thermo Fisher Scientific	S36963
RNase A, DNase and protease-free	Thermo Fisher Scientific	EN0531
RNaseZap	Thermo Fisher Scientific	AM9780
10X PBS	Invitrogen	AM9624
DEPC-Treated Water	Thermo Fisher Scientific	AM9920
RNAlater	Sigma-Aldrich	R0901
TRIzol Reagent	Invitrogen	15596026
Ethyl alcohol	Sigma-Aldrich	E7023
2-Mercaptoethanol	Sigma-Aldrich	M6250
QIAshredder	Qiagen	79654
Rneasy Mini Kit	Qiagen	74106
SuperScript IV First-Strand Synthesis System	Thermo Fisher	18091050
TaqMan Fast Advanced Master Mix for qPCR	Thermo Fisher Scientific	4444557
Molasses food	Archon Scientific Inc.	B70101
Qubit DNA HS Assay kit	Invitrogen	Q32854
Qubit RNA HS Assay kit	Invitrogen	Q32855
TURBO DNA-free Kit	Invitrogen	AM1907
**Fly strains**		
*w^1118^*	-	-
*TPN2-GAL4* (regular)	Kim *et al.* (*35*)	-
*TPN2-ss-GAL4*	Gift from B. Dickson	SS66351
*Earmuff*	Janelia	SS31063
*Tophat* (*1*)	Janelia	SS39993
*Tophat* (2)	Janelia	SS39932
*Peafowl*	Janelia	SS37792
*Amulet*	Janelia	SS32423
*Sullivan*	Janelia	SS42639
*Bobber*	Janelia	SS29032
*Turner*	Janelia	SS49422
*Kelp*	Janelia	SS43885
*Hound*	Janelia	SS47232
*UAS>STOP>KIR-eGFP*	Watanabe *et al.* (*36*)	-
*Otd-FLP*	Asahina *et al.* (*37*)	-
*UAS>stop>CsChrimson^tdt^*	Watanabe *et al.* (*36*)	-
*vGAT-lexA*	BDSC	BL-84441
*V5-VGAT-KI*	BDSC	BL-94893
*oviDN-ss-GAL4*	Janelia	SS46540
*oviEN-ss-GAL4*	Janelia	SS65426
*UAS-Kir2.1*	Baines *et al.* (*38*)	-
*Empty-ss-GAL4*	BDSC	BL-79603
*UAS-CsCrimson^venus^*	BDSC	BL-55136
*UAS-CsChrimson^tdT^ (x)*	Gift from T.H.	-
*lexA-CsChrimson^tdt^ (x)*	Gift from T.H.	-
*TPN2-LexA*	Gift from B. Dickson	*VT-057358-LexA*
*LexA-FLP*	BDSC	BL-55819
*LexA-CsChrimson^venus^*	BDSC	BL-55139
GRASP (*spGFPs*)	BDSC	BL-64315
*UAS-GCaMP6f*	BDSC	BL-52869
*UAS-GCaMP6s*	BDSC	BL-42746
Oahu, Hawaii (line 0)	Cornell Stock Center	14021-0231.00
Mysore, India (line 06)	Cornell Stock Center	14021-0231.06
Captain Cook, Hawaii (line 54)	Cornell Stock Center	14021-0231.54
Guam (line 198)	Cornell Stock Center	14021-0231.198
Kuala Lumpur, Malaysia (line 04)	Cornell Stock Center	14021-0231.04
Cape Town, South Africa (line 51)	Cornell Stock Center	14021-0231.51
Vinasco, Mexico (line 42)	Cornell Stock Center	14021-0231.42
Coffs Harbour, Australia (line 18)	Cornell Stock Center	14021-0231.18
Le Reduit, Mauritius (line 53)	Cornell Stock Center	14021-0231.53
*pum* allele, *P{lacW}pum^bem^*	BDSC (*39*)	BL-6782
*pum* allele, *pum[DeltaLCR1+LCR2]*	BDSC	BL-97221
*pum* allele, *Mi{ET1}pum^MB06187^*	BDSC	BL-25480
*UAS-pum-RNAi*	BDSC	BL-38241
*UAS-para*	Piggott *et al.* (*40*)	-
*UAS-nanos-RNAi*	BDSC	BL-28300
*UAS-pum*	Gift from J. Dubnau (*41*)	-
*UAS-GCaMP8m*	BDSC	BL-92592
*UAS-GCaMP7b*	BDSC	BL-79029
*UAS-TNT*	BDSC	BL-28996
*Gr5a^LexA^*	BDSC	BL-93440
*Gr64f^LexA^*	BDSC	BL-93445
*UAS-syt-eGFP*	BDSC	BL-33065
**Software**		
Automatic egg counter	In-house	https://doi.org/10.5281/zenodo.18839001
Motion-correction app	In-house	Based on NoRMCorre (*42*)
SkinnerTrax v1.0.2	Stern and Yang (*34*)	-
Prism 10	GraphPad	-
ImageJ/Fiji	https://imagej.net/ij/	-

**Table 2.** Genetic crosses and abbreviated genotypes for all experiments by figures. We note that crosses were not performed with a consistent choice of which parental strain provided virgin females versus males, except when we needed to selectively assay male progeny.

	Abbreviated crosses
Figure 1	
D and E	*w^1118^*
	*14021-0231.00* (Oahu, Hawaii)
	*14021-0231.53* (Mauritius)
	*14021-0231.06* (Mysore, India)
	*14021-0231.54* (Captain Cook, Hawaii)
	*14021-0231.198* (Guam)
	*14021-0231.04* (Kuala Lumpur, Malaysia), aka *04*
	*14021-0231.158* (Montpellier, France)
	*14021-0231.57* (Summerhaven, Arizona)
	*14021-0231.51* (Cape Town, South Africa), aka *51*
	*14021-0231.128* (Southwest Harbor, Maine), aka *128*
	*14021-0231.42* (Vinasco, Mexico), aka *42*
	*14021-0231.18* (Coffs Harbour, Australia)
F	*w^1118^*
	*51*
	*128*
	*w^1118^ x 51*
	*w^1118^ x 128*
G	*128*
	*51*
	*128 x 51*
**Figure 2**	
A	*w^1118^*
	*51*
C	*51/51* x *w^1118^/51*
F	*51 x 51*
	*w^1118^ x 51*
	*51 x pum* (BL-6782)
	*w^1118^ x pum* (BL-6782)
G	*w^1118^ x pum* (BL-6782)
	*51 x pum* (BL-6782)
	*51 x pum* (BL-97221)
	*51 x pum* (BL-25480)
H	*w^1118^ x pum* (BL-6782)
	*42 x pum* (BL-6782)
I	*42*
	*w^1118^*
J	*w^1118^ x pum* (BL-6782)
	*42 x pum* (BL-6782)
**Figure 3**	
A	*oviDN-ss-GAL4 x otd-FLP, UAS>stop>CsChrimson^tdt^*
	Note: *otd-FLP, UAS>stop>effector* was used to restrict effector expression to *GAL4*-labeled neurons in the brain.
B	*UAS-CsChrimson^tdt^; TPN2-ss-GAL4 x w^1118^*
	**Abbreviated crosses**
E	*Empty-ss-GAL4 x UAS-Kir*
	*Tophat x UAS-Kir*
	*Tophat_II x UAS-Kir*
	*Peafowl x UAS-Kir*
	*Amulet x UAS-Kir*
	*Earmuff x UAS-Kir*
	*Sullivan x UAS-Kir*
	*Bobber x UAS-Kir*
	*Turner x UAS-Kir*
	*Kelp x UAS-Kir*
	*Hound x UAS-Kir*
F	*Empty-ss-GAL4 x UAS>stop>Kir*
	*Earmuff-ss-GAL4 x Otd-FLP*
	*Earmuff-ss-GAL4 x Otd-FLP; UAS>stop>Kir*
J and K	*Empty-ss-GAL4 x otd-FLP, UAS>stop>CsChrimson^tdt^*
	*Earmuff-ss-GAL4 x otd-FLP, UAS>stop>CsChrimson^tdt^*
**Figure 4**	
A	*UAS>stop>GFP; Earmuff-ss-GAL4 x vGAT-LexA; LexA-FLP*
D	*Earmuff-ss-GAL4 x TPN2-LexA; spGFPs*
H and I	*Earmuff-ss-GAL4 x UAS-GCaMP6f*
**Figure 5**	
B	*UAS-GCaMP8m; Earmuff^ AD^/CyO; Earmuff ^DBD^, pum^BL-6782^/TM2 x w^1118^*
	*UAS-GCaMP8m; Earmuff^ AD^/CyO; Earmuff^ DBD^, pum^BL-6782^/TM2 x 51*
C	*UAS-GCaMP8s; Earmuff^ AD^/CyO; Earmuff^ DBD^, pum^BL-6782^/TM2 x w^1118^*
	*UAS-GCaMP8s; Earmuff^ AD^/CyO; Earmuff^ DBD^, pum^BL-6782^/TM2 x 42*
D	*UAS-GCaMP8m; Earmuff-ss-GAL4 x w^1118^*
	*UAS-GCaMP8m; Earmuff-ss-GAL4 x UAS-pum-RNAi*
E	*UAS-GCaMP8m; Earmuff-ss-GAL4 x w^1118^*
	*UAS-GCaMP8m; Earmuff-ss-GAL4 x UAS-pum-RNAi*
F	*UAS-GCaMP8m; Earmuff-ss-GAL4 x w^1118^*
	*UAS-GCaMP8m; Earmuff-ss-GAL4 x UAS-nos-RNAi*
G	*UAS-GCaMP8m; Earmuff-ss-GAL4 x w^1118^*
	*UAS-GCaMP8m; Earmuff-ss-GAL4 x UAS-nos-RNAi*
H	*UAS-GCaMP8m; Earmuff^ AD^/CyO; Earmuff^ DBD^, pum^BL-6782^/TM2 x 51*
	*UAS-GCaMP8m; Earmuff^ AD^, UAS-Pum/CyO; Earmuff^ DBD^, pum^BL-6782^/TM2 x 51*
I	*UAS-GCaMP8m; Earmuff^AD^/CyO; Earmuff^DBD^, pum^BL-6782^/TM2 x 51*
	*UAS-GCaMP8m; Earmuff^AD^, UAS-Pum/CyO; Earmuff^DBD^, pum^BL-6782^/TM2 x 51*
J	*UAS-GCaMP8m; Earmuff-ss-GAL4 x w^1118^*
	*UAS-GCaMP8m; Earmuff-ss-GAL4 x UAS-para*
K	*UAS-GCaMP8m; Earmuff-ss-GAL4 x w^1118^*
	*UAS-GCaMP8m; Earmuff-ss-GAL4 x UAS-para*
L and M	*otd-FLP, UAS>stop>CsChrimson^tdt^ x Earmuff-ss-GAL4*
N	*w^1118^*
O	*Earmuff-ss-GAL4 x UAS-GCaMP6f*
	**Abbreviated crosses**
P	*UAS-GCaMP8s; Earmuff^ AD^/CyO; Earmuff^ DBD^, pum^BL-6782^/TM2 x w^1118^*
	*UAS-GCaMP8s; Earmuff^ AD^/CyO; Earmuff^ DBD^, pum^BL-6782^/TM2 x 51*
	*UAS-GCaMP8m; Earmuff-ss-GAL4 x w^1118^*
	*UAS-GCaMP8m; Earmuff-ss-GAL4 x UAS-pum-RNAi*
	*UAS-GCaMP8m; Earmuff-ss-GAL4 x w^1118^*
	*UAS-GCaMP8m; Earmuff-ss-GAL4 x UAS-para*
**Figure S2**	
A	*42*
	*51*
	*42 x 51*
B	*42*
	*128*
	*128 x 42*
C	*04*
	*51*
	*04 x 51*
D	*04*
	*128*
	*04 x 128*
E	*128*
	*51*
	*128 x 51*
F	*51*
	*53*
	*128*
**Figure S3**	
A	*UAS-CsChrimson^tdt^; TPN2-ss-GAL4 x w^1118^*
B	*UAS>stop>GFP; TPN2-ss-GAL4 x hs-FLP*
C	*TPN2-ss-GAL4 x Gr5a^LexA^; spGFPs*
D	*UAS>stop>GFP; TPN2-ss-GAL4 x TPN2-LexA; LexA-FLP*
E	*TPN2-LexA, UAS>stop>Kir; LexA-FLP x TPN2-ss-GAL4*
F	*TPN2-ss-GAL4 x UAS-GCaMP6s*
G	*LexA-CsChrimson^tdt^; UAS-GCaMP6s; Gr64f^ LexA^ x TPN2-ss-GAL4*
**Figure S4**	
A and A′	*Earmuff x UAS-GCaMP6f*
B	*lexA-CsChrimson^tdt^; Gr5a-lexA; UAS-GCaMP6f x Earmuff-ss-GAL4*
	*lexA-CsChrimson^tdt^; UAS-GCaMP6f x Earmuff*
C	*UAS-GCaMP8m; Earmuff x UAS-TNT; Empty-ss-GAL4*
	*UAS-GCaMP8m; Earmuff x UAS-TNT; TPN2-ss-GAL4*
D	*UAS-GCaMP8s; oviEN-ss-GAL4 x UAS-TNT; Empty-ss-GAL4*
	*UAS-GCaMP8s; oviEN-ss-GAL4 x UAS-TNT; Earmuff-ss-GAL4*
E	*BL-94893 (v5-vGAT-KI) X Earmuff*
**Figure S5**	
A	*otd-FLP, UAS>stop>CsChrimson^tdt^ x Earmuff-ss-GAL4*
B	*TPN2-lexA; SpGFPs x Earmuff-ss-GAL4*
	**Abbreviated crosses**
**Figure S6**	
A to A″	*UAS-GCaMP8m;Earmuff^ AD^; Earmuff^ DBD^, pum^BL-6782^/TM2 x w^1118^*
	*UAS-GCaMP8m;Earmuff^ AD^; Earmuff^ DBD^, pum^BL-6782^/TM2 x 51*
B to B″	*UAS-GCaMP8s;Earmuff^ AD^; Earmuff^ DBD^, pum^BL-6782^/TM2 x w^1118^*
	*UAS-GCaMP8s;Earmuff^ AD^; Earmuff^ DBD^, pum^BL-6782^/TM2 x 42*
C	*UAS-GCaMP8s;Earmuff^ AD^; Earmuff^ DBD^, pum^BL-6782^/TM2 x w^1118^*
	*UAS-GCaMP8s;Earmuff^ AD^; Earmuff^ DBD^, pum^BL-6782^/TM2 x 42*
**Figure S7**	
A to A″	*UAS-GCaMP8m; Earmuff-ss-GAL4x w^1118^*
	*UAS-GCaMP8m; Earmuff-ss-GAL4 x UAS-pum-RNAi*
B to B″	*UAS-GCaMP8m; Earmuff-ss-GAL4 x w^1118^*
	*UAS-GCaMP8m; Earmuff-ss-GAL4 x UAS-nos-RNAi*
C to C″	*UAS-GCaMP8m; Earmuff^ AD^/CyO; Earmuff^ DBD^, pum^BL-6782^/TM2 x 51*
	*UAS-GCaMP8m; Earmuff^ AD^, UAS-Pum/CyO; Earmuff^ DBD^, pum^BL-6782^/TM2 x 51*
D to D″	*UAS-GCaMP8m; Earmuff-ss-GAL4 X w^1118^*
	*UAS-GCaMP8m; Earmuff-ss-GAL4 X UAS-para*
**Figure S8**	
A and A′	*TPN2-ss-GAL4 x UAS-GCaMP6s*
B	*TPN2-ss-GAL4 x UAS-pum-RNAi*
	*Empty-ss-GAL4 x UAS-pum-RNAi*
	*Empty-ss-GAL4 x UAS-para*
	*TPN2-ss-GAL4 x UAS-para*
C to E	*UAS-GCaMP6s; TPN2-GAL4, pum^BL-6782^ x w^1118^*
	*UAS-GCaMP6s; TPN2-GAL4, pum^BL-6782^ x 51*
F to H	*TPN2^AD^, UAS-GCaMP7b; TPN2^DBD^ x w1118*
	*TPN2^AD^, UAS-GCaMP7b; TPN2^DBD^ x UAS-pum-RNAi*
I to K	*UAS-GCaMP6s; TPN2-GAL4 x w^1118^*
	*UAS-GCaMP6s; TPN2-GAL4 x UAS-para*

### Immunostaining

Brains and ventral nerve cords (VNCs) were dissected and fixed in 4% paraformaldehyde prepared in phosphate-buffered saline (PBS) containing 0.3% Triton X-100 (PBST). The dilution factors for the antibodies used in this study were as follows: NC82: 1:50, anti-GFP: 1:1000, anti-RFP: 1:5000, anti-V5: 1:500, donkey anti-mouse Cy3: 1:500, donkey anti-mouse Alexa Fluor 488: 1:500, donkey anti-rabbit Alexa Fluor 594: 1:500, and donkey anti-rabbit Alexa Fluor 488: 1:500. Samples were imaged using a Zeiss LSM700 with either a 20x air objective or a 40x oil objective, and the acquired confocal images were then postprocessed with Fiji. For reagents used, see Table 1.

### Reverse transcription quantitative polymerase chain reaction

RNA extraction: Female fly brains (*n* = 30) or heads (*n* = 15) were dissected in RNase-free PBS and transferred to TRIzol reagent. RNA was extracted using the Qiagen RNeasy Kit and Qiagen QIAshredder column, following the manufacturer’s protocol. DNase treatment: To remove genomic DNA contamination, samples were treated with TURBO DNase. RNA concentration was measured using the Qubit RNA High Sensitivity Assay Kit. cDNA synthesis: cDNA was synthesized from total RNA using the SuperScript IV First-Strand Synthesis System, following the manufacturer’s instructions. Reverse transcription quantitative polymerase chain reaction (RT-qPCR): We used a QuantStudio 3 to perform qPCR using TaqMan Fast Advanced Master Mix, following the manufacturer’s protocol. Gene expression levels were assessed using TaqMan Gene Expression Assays with Dm02361909_s1 (*act5C*, housekeeping gene) and Dm02135527_m1 (that targets all isoforms of *pum*). For reagents used, see Table 1.

### Full-genome sequencing SNP analysis

DNA extraction: We extracted DNA from individual F2 recombinants using a standard protocol (*31*). Whole-genome sequencing: We used SNPsaurus and Novogene to perform whole-genome sequencing of a total of 192 of the selected F2 recombinants. SNPsaurus and Novogene sequenced 96 flies each using Illumina NovaSeq sequencers, with paired-end reads of 159 and 150 base pairs (bp), respectively, and coverages of 10x and 20x, respectively. Variant calling pipeline: We implemented our own GATK-based variant (SNP and indel) calling pipeline to turn the raw reads (FASTQ files) into a VCF file that gives the variant calls for each fly. The pipeline used GATK tools when available, but we preferred fastp to MarkIlluminaAdapters to take advantage of fastp’s adapter autodetection. We also wrote a Python script to run pipelines in parallel, making efficient use of modern CPUs’ many cores. For both the pipeline and the script, we strove for low SSD wear. Our pipeline produced per-sample GVCF files, which we combined into a single “cohort” VCF file for all 192 flies. We filtered out genotype calls in the VCF file with genotype quality (GQ) below 13, corresponding to a 5% call error probability. GWAS using PLINK. The goal of our GWAS using PLINK (*24*) was to identify variants associated with sucrose acceptance. We used the flies’ PI both as a quantitative trait and to group flies into “sucrose accepting” and “sucrose rejecting” flies (case/control phenotype). For the group-based analysis, we ran the analysis twice with different thresholds: first for groups “PI ≥ −0.1” and “PI ≤ −0.75” (with 49 and 82 flies, respectively) and then for groups “PI ≥ 0” and “PI ≤ −0.80” (with 38 and 73 flies, respectively). We used PLINK’s max(T) permutation procedure with 5 million permutations and Fisher’s exact test to calculate multiple-comparison-corrected empirical *P* values (EMP2). We next performed a quantitative trait analysis using PI as a continuous variable. From this analysis, we specifically selected the genomic control-corrected *P* values (GC) and the Bonferroni-corrected *P* values (BONF). In table S1, we show the 19 variants from the cohort VCF file that were significantly associated (*P* < 0.05) with sucrose acceptance or PI simultaneously under each of the four adjusted *P* value calculations: EMP2 (one for each grouping), GC, and BONF. To identify genes potentially affected by each of the 19 variants, we used SnpEff, and the rightmost column in table S1 shows those genes and the relative location of the variant. Another column lists the beta values PLINK computed as part of the quantitative trait analysis. The beta values can be used to estimate effect size and were calculated by fitting (for each variant) PI = α + β·*G* + ε, with *G* being the number of copies of the A1 allele. Because the inclusion criterion for table S1 was very strict (significance under each of the four adjusted *P* values), we also added table S3 with relaxed inclusion, which has 336 variants. VCF files are available to be shared with readers interested in performing their own analyses.

### Egg-laying assay (standard)

Preparation of flies for the assay: We used our egg-laying deprivation protocol previously described (*32*). Briefly, we grouped 35 females with ~10 to 15 males in a food vial supplemented with yeast paste (live yeast granules mixed with 0.5% propionic acid) for 4 days. This was to induce females to produce and maintain many eggs in an environment that lacks a good surface for egg laying due to an excess of larvae by day 4. As such, females will readily lay eggs when placed in our custom chambers (apparatuses). To load flies into the arena, we anesthetized them with CO_2_ and kept exposure under 2 min. After loading, flies recovered for at least 15 min before substrate access (i.e., before the portion of the chamber housing the flies initially and the plate holding the substrates were assembled.) Preparation of egg-laying substrates: We prepared substrates immediately before loading individual females (deprived using the protocol described above) into our custom chambers. Sucrose substrates (150 mM sucrose) were prepared by diluting 2 M sucrose stock into 1% agarose (e.g., 750 μl of 2 M stock into 10 ml of 1% agarose). Plain substrates were prepared by adding the same volume of ddH_2_O to 1% agarose. We routinely keep a bottle of molten 1% agarose in a 55°C water bath. After loading, flies were covered and incubated at 25°C and 60% humidity for egg laying. Egg counting: After allowing flies to lay eggs for 18 to 20 hours (overnight), we placed the bottom plate on a light pad and acquired an 18-megapixel image using a stand-mounted and leveled Canon EOS Rebel T3i (fig. S1). We then used a custom-built automated egg-counting tool to segment arenas and count the eggs laid in each trough. Code and performance benchmarks are available at https://doi.org/10.5281/zenodo.18839001. The tool is a web-based application that automatically quantifies eggs in each agarose region from an overhead photograph of the bottom plate of a multifly egg-laying chamber. After the user uploads an image, a neural network detects the circular hole at the center of each fly enclosure (arena) and uses these holes as anchor points to define segmentation boxes around the two agarose regions at opposite ends of each arena. Each agarose region is then cropped into its own subimage, which is passed to a second neural network trained to detect individual eggs. Last, the user can review the automatically identified eggs, correct counts if needed, and download the results either as a CSV (with egg counts per agarose region) or as a fully annotated image. Calculation of PI: We then calculated the PI for each animal using the following formula: (# of eggs on sucrose − # of eggs on plain)/(total # of eggs). This index reflects the relative preference for sucrose over plain agarose. If a given fly laid fewer than 10 eggs, its PI was omitted for analysis—we adopted the view that flies’ preferences are clearer if they had laid at least 10 eggs.

### Egg-laying assay (optogenetics)

Fly and chamber preparations: Female flies were prepared and their egg-laying PI was calculated as in the nonoptogenetics case, but in the optogenetics case, vials were kept in the dark and the food was supplemented with 200 mM all-*trans*-retinal to allow *CsChrimson* to be activated by light. In addition, plain agarose substrates were supplemented with 100 mM sorbitol to ensure flies had access to nutrition during the assay. Egg-laying assay: Flies were loaded into arenas under dim lighting conditions and were allowed to lay eggs overnight (>14 hours) in the SkinnerSys setup, a high-throughput closed-loop stimulation platform. The setup included several custom components optimized for tracking and stimulation: (i) SkinnerTrax: custom software for real-time tracking of multiple flies while delivering light pulses to individual flies based on their behaviors (*33*, *34*); (ii) modified two-choice arenas with shorter and slightly angled sidewalls; (iii) light-emitting diode (LED) stimulation setup: a custom printed circuit board (PCB) equipped with 80 individually controllable red (624 nm) Cree LEDs, with two LEDs per arena for uniform illumination; (iv) apparatus stand: a custom-designed stand that held the arenas, cameras (Microsoft LifeCam Cinema), and PCB in fixed positions. A dimmed light pad (Artograph LightPad A920) provided low-intensity backlighting for tracking without activating channelrhodopsins. Cameras were fitted with blue-pass filters (LEE Filters 172 Lagoon Blue) to reduce the pulsing red LEDs’ impact on tracking. Stimulation protocol: Optogenetic stimulation was applied only when SkinnerTrax detected that a fly was positioned over the agarose substrate of interest. The LEDs for that arena were pulsed at 2 Hz (250-ms on/off), providing ~6.8 μW/mm^2^ of red light during “LED on.”

### Calcium imaging

Preparation: Each fly was anesthetized individually on a CO_2_ pad for less than 2 min, and the head was positioned through an opening in an aluminum foil plate. Ultraviolet-cured glue was applied to immobilize its wings and thorax, whereas the eyes and proboscis were also secured. The head was then submerged in 1X standard imaging buffer (*21*) freshly supplemented with 4 mM CaCl_2_. A small section of the cuticle was carefully removed to expose the region of interest (ROI). An additional 100 μl of 1X imaging solution with 4 mM CaCl_2_ was applied to facilitate imaging with a water immersion objective. Image acquisition: Imaging was performed at 25°C using a ZEISS LSM700 laser confocal microscope equipped with a 40X water immersion objective, at 128 by 128 pixels and 8 fps. Stimulation protocol for substrate contacts: During imaging, agarose with 150 mM sucrose or plain agarose was presented to the fly’s legs for about 60 s using a micromanipulator. Each substrate was presented three times in alternating order with about 30 s of no substrate in between agarose presentations. Stimulation protocol for solution contacts: To acclimate the fly’s legs to moisture, 100 μl of deionized (DI) water was first applied, ensuring that subsequent response to the sucrose solution would be specifically due to sugar detection by the leg chemosensory receptors rather than a response to moisture. Following this acclimation step, 50 μl of 450 mM either sucrose or sorbitol was added to the 100 μl of DI water in which the fly’s legs were submerged. Fluorescence quantification and analysis: Raw LSM files were first uploaded to an in-house application for motion correction to improve accuracy of fluorescence measurements. ROIs were defined in ImageJ to measure fluorescence intensity. For Earmuff neurons, the ROI was drawn around the soma in each hemisphere, whereas for TPN2 neurons, the ROI encompassed the arbors within the SEZ region. For oviDN and oviEN neurons, the ROI was drawn in the SMP region of the brain. Baseline fluorescence (*F*_0_) for each substrate was determined from 4 to 10 time points immediately preceding leg contact with the substrate, depending on the experiment. Fluorescence values [*F*(*t*)] were then measured, and the change in fluorescence (Δ*F*/*F*) was calculated using the formula: Δ*F*/*F* = (*F*(*t*) − *F*_0_)/*F*_0_. Δ*F*/*F* values were calculated over the entire duration of substrate contact and averaged across three trials for each substrate condition.

### Statistics

All statistical tests, except the SNP analysis, were performed using GraphPad Prism 10. Sample sizes are stated in the figure legends. Unless mentioned otherwise, each dot in a swarm plot and each sample represents a biological replicate (an individual fly). For Ca^2+^ imaging, we typically averaged responses from three technical replicates for the same fly to give one sample. Bars and whiskers indicate mean and 95% confidence interval (CI). Similarly, Δ*F*/*F* graphs show mean and 95% CI bands (e.g., Fig. 4H). Also, we used unpaired *t* tests with Welch’s correction to compare two samples, except for within-fly comparisons (e.g., Fig. 4I) where paired *t* tests were used, and one-way analysis of variance (ANOVA) (with Tukey’s multiple comparisons test) to compare three or more samples. To assess whether a sample mean was different from 0, we used one-sample *t* tests. All tests were two sided. The following notation is used to indicate *P* value ranges but is not meant as a strict “significant/nonsignificant” dichotomy: n.s. (not significant): *P* ≥ 0.05, *P < 0.05, **P < 0.01, ***P < 0.001, and ****P < 0.0001.

## References

[1] M. de Bono, C. I. Bargmann, Natural variation in a neuropeptide Y receptor homolog modifies social behavior and food response in *C. elegans*. Cell 94, 679–689 (1998). 9741632 10.1016/s0092-8674(00)81609-8

[2] A. Wada-Katsumata, J. Silverman, C. Schal, Changes in taste neurons support the emergence of an adaptive behavior in cockroaches. Science 340, 972–975 (2013). 23704571 10.1126/science.1234854

[3] M. P. Shahandeh, L. Abuin, L. Lescuyer De Decker, J. Cergneux, R. Koch, E. Nagoshi, R. Benton, Circadian plasticity evolves through regulatory changes in a neuropeptide gene. Nature 635, 951–959 (2024). 39415010 10.1038/s41586-024-08056-x PMC11602725

[4] M. M. Lim, Z. Wang, D. E. Olazabal, X. Ren, E. F. Terwilliger, L. J. Young, Enhanced partner preference in a promiscuous species by manipulating the expression of a single gene. Nature 429, 754–757 (2004). 15201909 10.1038/nature02539

[5] M. N. Shadlen, R. Kiani, Decision making as a window on cognition. Neuron 80, 791–806 (2013). 24183028 10.1016/j.neuron.2013.10.047 PMC3852636

[6] M. N. Shadlen, W. T. Newsome, Motion perception: Seeing and deciding. Proc. Natl. Acad. Sci. U.S.A. 93, 628–633 (1996). 8570606 10.1073/pnas.93.2.628 PMC40102

[7] D. Ottenheimer, J. M. Richard, P. H. Janak, Ventral pallidum encodes relative reward value earlier and more robustly than nucleus accumbens. Nat. Commun. 9, 4350 (2018). 30341305 10.1038/s41467-018-06849-z PMC6195583

[8] C. Padoa-Schioppa, J. A. Assad, Neurons in the orbitofrontal cortex encode economic value. Nature 441, 223–226 (2006). 16633341 10.1038/nature04676 PMC2630027

[9] K. M. Cury, B. Prud’homme, N. Gompel, A short guide to insect oviposition: When, where and how to lay an egg. J. Neurogenet. 33, 75–89 (2019). 31164023 10.1080/01677063.2019.1586898

[10] C. H. Yang, P. Belawat, E. Hafen, L. Y. Jan, Y. N. Jan, Drosophila egg-laying site selection as a system to study simple decision-making processes. Science 319, 1679–1683 (2008). 18356529 10.1126/science.1151842 PMC2581776

[11] C. H. Yang, R. He, U. Stern, Behavioral and circuit basis of sucrose rejection by Drosophila females in a simple decision-making task. J. Neurosci. 35, 1396–1410 (2015). 25632118 10.1523/JNEUROSCI.0992-14.2015 PMC4308591

[12] V. Vijayan, Z. Wang, V. Chandra, A. Chakravorty, R. Li, S. L. Sarbanes, H. Akhlaghpour, G. Maimon, An internal expectation guides Drosophila egg-laying decisions. Sci. Adv. 8, eabn3852 (2022). 36306348 10.1126/sciadv.abn3852 PMC9616500

[13] V. Vijayan, F. Wang, K. Wang, A. Chakravorty, A. Adachi, H. Akhlaghpour, B. J. Dickson, G. Maimon, A rise-to-threshold process for a relative-value decision. Nature 619, 563–571 (2023). 37407812 10.1038/s41586-023-06271-6 PMC10356611

[14] F. Wang, K. Wang, N. Forknall, C. Patrick, T. Yang, R. Parekh, D. Bock, B. J. Dickson, Neural circuitry linking mating and egg laying in Drosophila females. Nature 579, 101–105 (2020). 32103180 10.1038/s41586-020-2055-9 PMC7687045

[15] S. Dorkenwald, A. Matsliah, A. R. Sterling, P. Schlegel, S.-C. Yu, C. E. McKellar, A. Lin, M. Costa, K. Eichler, Y. Yin, W. Silversmith, C. Schneider-Mizell, C. S. Jordan, D. Brittain, A. Halageri, K. Kuehner, O. Ogedengbe, R. Morey, J. Gager, K. Kruk, E. Perlman, R. Yang, D. Deutsch, D. Bland, M. Sorek, R. Lu, T. Macrina, K. Lee, J. A. Bae, S. Mu, B. Nehoran, E. Mitchell, S. Popovych, J. Wu, Z. Jia, M. A. Castro, N. Kemnitz, D. Ih, A. S. Bates, N. Eckstein, J. Funke, F. Collman, D. D. Bock, G. S. X. E. Jefferis, H. S. Seung, M. Murthy, FlyWire Consortium, Neuronal wiring diagram of an adult brain. Nature 634, 124–138 (2024). 39358518 10.1038/s41586-024-07558-y PMC11446842

[16] L. K. Scheffer, C. S. Xu, M. Januszewski, Z. Lu, S.-Y. Takemura, K. J. Hayworth, G. B. Huang, K. Shinomiya, J. Maitlin-Shepard, S. Berg, J. Clements, P. M. Hubbard, W. T. Katz, L. Umayam, T. Zhao, D. Ackerman, T. Blakely, J. Bogovic, T. Dolafi, D. Kainmueller, T. Kawase, K. A. Khairy, L. Leavitt, P. H. Li, L. Lindsey, N. Neubarth, D. J. Olbris, H. Otsuna, E. T. Trautman, M. Ito, A. S. Bates, J. Goldammer, T. Wolff, R. Svirskas, P. Schlegel, E. Neace, C. J. Knecht, C. X. Alvarado, D. A. Bailey, S. Ballinger, J. A. Borycz, B. S. Canino, N. Cheatham, M. Cook, M. Dreher, O. Duclos, B. Eubanks, K. Fairbanks, S. Finley, N. Forknall, A. Francis, G. P. Hopkins, E. M. Joyce, S. Kim, N. A. Kirk, J. Kovalyak, S. A. Lauchie, A. Lohff, C. Maldonado, E. A. Manley, S. McLin, C. Mooney, M. Ndama, O. Ogundeyi, N. Okeoma, C. Ordish, N. Padilla, C. M. Patrick, T. Paterson, E. E. Phillips, E. M. Phillips, N. Rampally, C. Ribeiro, M. K. Robertson, J. T. Rymer, S. M. Ryan, M. Sammons, A. K. Scott, A. L. Scott, A. Shinomiya, C. Smith, K. Smith, N. L. Smith, M. A. Sobeski, A. Suleiman, J. Swift, S. Takemura, I. Talebi, D. Tarnogorska, E. Tenshaw, T. Tokhi, J. J. Walsh, T. Yang, J. A. Horne, F. Li, R. Parekh, P. K. Rivlin, V. Jayaraman, M. Costa, G. S. Jefferis, K. Ito, S. Saalfeld, R. George, I. A. Meinertzhagen, G. M. Rubin, H. F. Hess, V. Jain, S. M. Plaza, A connectome and analysis of the adult *Drosophila* central brain. Elife 9, e57443 (2020). 32880371 10.7554/eLife.57443 PMC7546738

[17] P. Schlegel, Y. Yin, A. S. Bates, S. Dorkenwald, K. Eichler, P. Brooks, D. S. Han, M. Gkantia, M. Dos Santos, E. J. Munnelly, G. Badalamente, L. Serratosa Capdevila, V. A. Sane, A. M. C. Fragniere, L. Kiassat, M. W. Pleijzier, T. Sturner, I. F. M. Tamimi, C. R. Dunne, I. Salgarella, A. Javier, S. Fang, E. Perlman, T. Kazimiers, S. R. Jagannathan, A. Matsliah, A. R. Sterling, S. C. Yu, C. E. McKellar, FlyWire Consortium, M. Costa, H. S. Seung, M. Murthy, V. Hartenstein, D. D. Bock, G. Jefferis, Whole-brain annotation and multi-connectome cell typing of *Drosophila*. Nature 634, 139–152 (2024). 39358521 10.1038/s41586-024-07686-5 PMC11446831

[18] R. P. Wharton, J. Sonoda, T. Lee, M. Patterson, Y. Murata, The Pumilio RNA-binding domain is also a translational regulator. Mol. Cell 1, 863–872 (1998). 9660969 10.1016/s1097-2765(00)80085-4

[19] P. D. Zamore, J. R. Williamson, R. Lehmann, The Pumilio protein binds RNA through a conserved domain that defines a new class of RNA-binding proteins. RNA 3, 1421–1433 (1997). 9404893 PMC1369583

[20] C. J. Mee, E. C. Pym, K. G. Moffat, R. A. Baines, Regulation of neuronal excitability through pumilio-dependent control of a sodium channel gene. J. Neurosci. 24, 8695–8703 (2004). 15470135 10.1523/JNEUROSCI.2282-04.2004 PMC6729971

[21] H.-L. Chen, D. Motevalli, U. Stern, C.-H. Yang, A functional division of *Drosophila* sweet taste neurons that is value-based and task-specific. Proc. Natl. Acad. Sci. U.S.A. 119, e2110158119 (2022). 35031566 10.1073/pnas.2110158119 PMC8784143

[22] J. L. Myers, M. Porter, N. Narwold, K. Bhat, B. Dauwalder, G. Roman, Mutants of the white ABCG transporter in *Drosophila melanogaster* have deficient olfactory learning and cholesterol homeostasis. Int. J. Mol. Sci. 22, 12967 (2021). 34884779 10.3390/ijms222312967 PMC8657504

[23] J. Borycz, J. A. Borycz, A. Kubow, V. Lloyd, I. A. Meinertzhagen, Drosophila ABC transporter mutants white, brown and scarlet have altered contents and distribution of biogenic amines in the brain. J. Exp. Biol. 211, 3454–3466 (2008). 18931318 10.1242/jeb.021162

[24] C. C. Chang, C. C. Chow, L. C. Tellier, S. Vattikuti, S. M. Purcell, J. J. Lee, Second-generation PLINK: Rising to the challenge of larger and richer datasets. Gigascience 4, 7 (2015). 25722852 10.1186/s13742-015-0047-8 PMC4342193

[25] G. R. Sterne, H. Otsuna, B. J. Dickson, K. Scott, Classification and genetic targeting of cell types in the primary taste and premotor center of the adult *Drosophila* brain. Elife 10, e71679 (2021). 34473057 10.7554/eLife.71679 PMC8445619

[26] N. C. Klapoetke, Y. Murata, S. S. Kim, S. R. Pulver, A. Birdsey-Benson, Y. K. Cho, T. K. Morimoto, A. S. Chuong, E. J. Carpenter, Z. Tian, J. Wang, Y. Xie, Z. Yan, Y. Zhang, B. Y. Chow, B. Surek, M. Melkonian, V. Jayaraman, M. Constantine-Paton, G. K. Wong, E. S. Boyden, Independent optical excitation of distinct neural populations. Nat. Methods 11, 338–346 (2014). 24509633 10.1038/nmeth.2836 PMC3943671

[27] S. R. Walker, M. Pena-Garcia, A. V. Devineni, Connectomic analysis of taste circuits in Drosophila. bioRxiv 2024.09.14.613080 [Preprint] (2024). 10.1101/2024.09.14.613080. PMC11821855 39939650

[28] J. Sonoda, R. P. Wharton, Recruitment of Nanos to hunchback mRNA by Pumilio. Genes Dev. 13, 2704–2712 (1999). 10541556 10.1101/gad.13.20.2704 PMC317116

[29] N. I. Muraro, A. J. Weston, A. P. Gerber, S. Luschnig, K. G. Moffat, R. A. Baines, Pumilio binds para mRNA and requires Nanos and Brat to regulate sodium current in Drosophila motoneurons. J. Neurosci. 28, 2099–2109 (2008). 18305244 10.1523/JNEUROSCI.5092-07.2008 PMC2323674

[30] N. U. Schwartz, L. Zhong, A. Bellemer, W. D. Tracey, Egg laying decisions in Drosophila are consistent with foraging costs of larval progeny. PLOS ONE 7, e37910 (2012). 22693584 10.1371/journal.pone.0037910 PMC3365076

[31] A. M. Huang, E. J. Rehm, G. M. Rubin, Quick preparation of genomic DNA from Drosophila. Cold Spring Harb. Protoc. 2009, pdb.prot5198 (2009). 20147141 10.1101/pdb.prot5198

[32] B. Gou, E. Zhu, R. He, U. Stern, C.-H. Yang, High throughput assay to examine egg-laying preferences of individual Drosophila melanogaster. J. Vis. Exp. 109, e53716 (2016). 10.3791/53716 PMC4841307 27077482

[33] U. Stern, H. Srivastava, H. L. Chen, F. Mohammad, A. Claridge-Chang, C. H. Yang, Learning a spatial task by trial and error in *Drosophila*. Curr. Biol. 29, 2517–2525.e5 (2019). 31327716 10.1016/j.cub.2019.06.045

[34] U. Stern, C.-H. Yang, SkinnerTrax: High-throughput behavior-dependent optogenetic stimulation of *Drosophila*. bioRxiv 080614 [Preprint] (2017). 10.1101/080614.

[35] H. Kim, C. Kirkhart, K. Scott, Long-range projection neurons in the taste circuit of *Drosophila*. Elife 6, e23386 (2017). 28164781 10.7554/eLife.23386 PMC5310837

[36] K. Watanabe, H. Chiu, B. D. Pfeiffer, A. M. Wong, E. D. Hoopfer, G. M. Rubin, D. J. Anderson, A circuit node that integrates convergent input from neuromodulatory and social behavior-promoting neurons to control aggression in Drosophila. Neuron 95, 1112–1128.e7 (2017). 28858617 10.1016/j.neuron.2017.08.017 PMC5588916

[37] K. Asahina, K. Watanabe, B. J. Duistermars, E. Hoopfer, C. R. Gonzalez, E. A. Eyjolfsdottir, P. Perona, D. J. Anderson, Tachykinin-expressing neurons control male-specific aggressive arousal in Drosophila. Cell 156, 221–235 (2014). 24439378 10.1016/j.cell.2013.11.045 PMC3978814

[38] R. A. Baines, J. P. Uhler, A. Thompson, S. T. Sweeney, M. Bate, Altered electrical properties in Drosophila neurons developing without synaptic transmission. J. Neurosci. 21, 1523–1531 (2001). 11222642 10.1523/JNEUROSCI.21-05-01523.2001 PMC6762927

[39] B. A. Schweers, K. J. Walters, M. Stern, The Drosophila melanogaster translational repressor pumilio regulates neuronal excitability. Genetics 161, 1177–1185 (2002). 12136020 10.1093/genetics/161.3.1177 PMC1462161

[40] B. J. Piggott, C. J. Peters, Y. He, X. Huang, S. Younger, L. Y. Jan, Y. N. Jan, Paralytic, the Drosophila voltage-gated sodium channel, regulates proliferation of neural progenitors. Genes Dev. 33, 1739–1750 (2019). 31753914 10.1101/gad.330597.119 PMC6942049

[41] G. Chen, W. Li, Q. S. Zhang, M. Regulski, N. Sinha, J. Barditch, T. Tully, A. R. Krainer, M. Q. Zhang, J. Dubnau, Identification of synaptic targets of Drosophila pumilio. PLOS Comput. Biol. 4, e1000026 (2008). 18463699 10.1371/journal.pcbi.1000026 PMC2265480

[42] E. A. Pnevmatikakis, A. Giovannucci, NoRMCorre: An online algorithm for piecewise rigid motion correction of calcium imaging data. J. Neurosci. Methods 291, 83–94 (2017). 28782629 10.1016/j.jneumeth.2017.07.031

