# Supplementary material for: Natural noncoding pumilio variants retune value-coding interneurons to bias Drosophila oviposition decisions
Source: Sci Adv. 2026 May 8;12(19):eaed1338. doi: 10.1126/sciadv.aed1338 (PMC13155294; doi:10.1126/sciadv.aed1338)
Supplement: Supplementary file 1 — Figs. S1 to S8 Table S1 Legends for tables S2 and S3 [file sciadv.aed1338_sm.pdf]

Science Advances
AAAS

Supplementary Materials for

Natural noncoding *pumilio* variants retune value-coding interneurons to bias *Drosophila* oviposition decisions

Dorsa Motevalli *et al.*

Corresponding author: Chung-Hui Yang, rebecca.yang@gmail.com

Sci. Adv. **12**, eaed1338 (2026)
DOI: 10.1126/sciadv.aed1338

The PDF file includes:

Figs. S1 to S8
Table S1
Legends for tables S2 and S3

Other Supplementary Material for this manuscript includes the following:

Tables S2 and S3

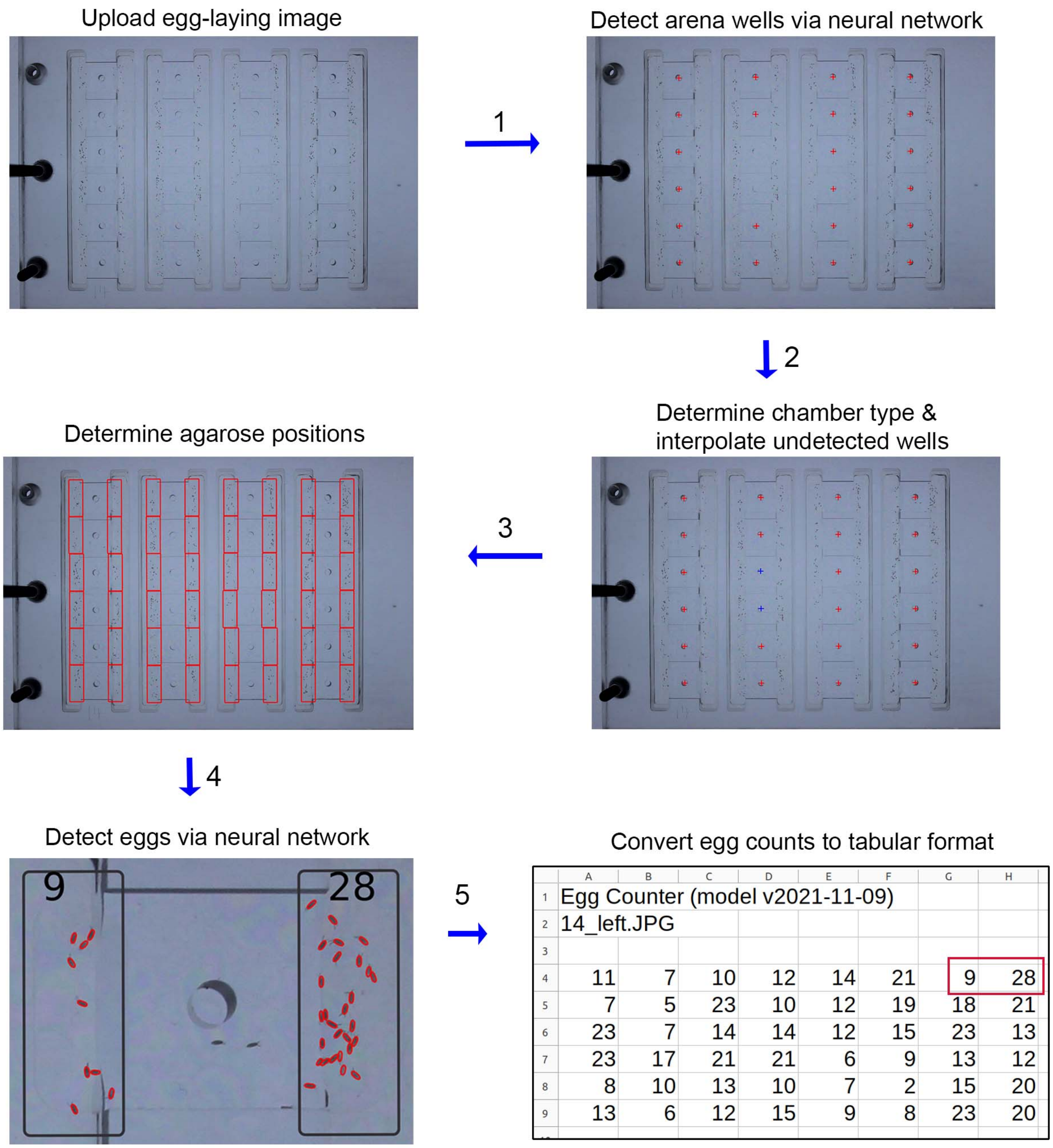

Figure S1. Automated egg-counting workflow.
A custom web application processes a base-plate image and returns egg counts for every arena: **1** Detect the central well in each arena with a convolutional neural network (CNN). **2** Identify chamber type and interpolate any undetected wells. **3** Segment the two agarose substrates per arena. **4** Detect eggs on each substrate with a second CNN. **5** Export counts to a CSV file.

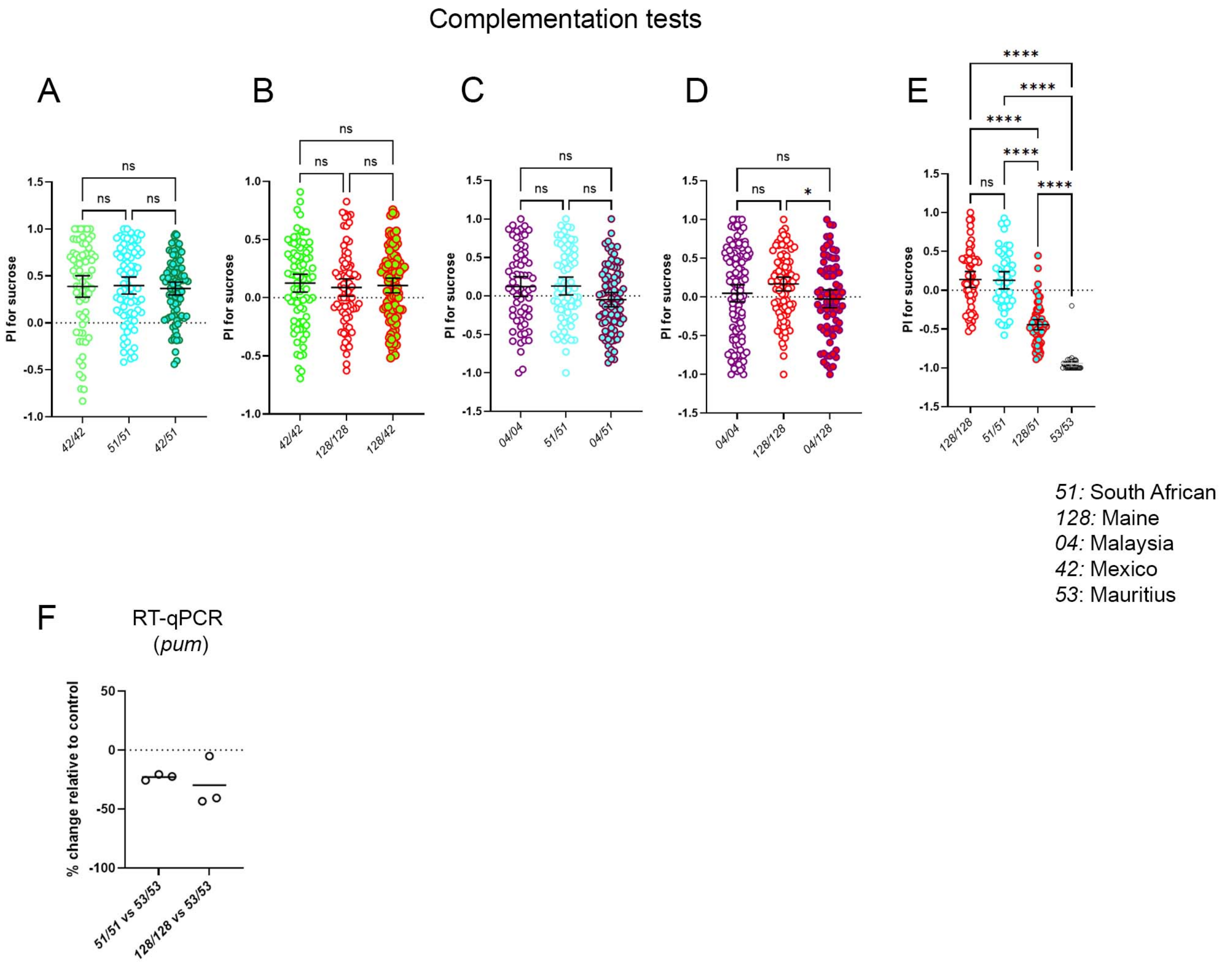

Figure S2. Complementation tests of additional wild-caught strains.
(**A** to **E**) PIs of the indicated parental lines and their F_1 progeny.
(**F**) RT–qPCR shows reduced *pum* expression in lines 51 and 128 relative to sucrose-rejecting wild-caught line 53; dots represent biological replicates.

Statistical tests in (**A** to **E**) were performed using one-way ANOVA followed by Tukey's multiple comparisons test. Expression levels in (**F**) were compared using unpaired two-sided Welch's t-tests. Significance is denoted as follows: ns: $p \geq 0.05$, *: $p < 0.05$, **: $p < 0.01$, ***: $p < 0.001$, and ****: $p < 0.0001$; all error bars represent 95% confidence intervals.

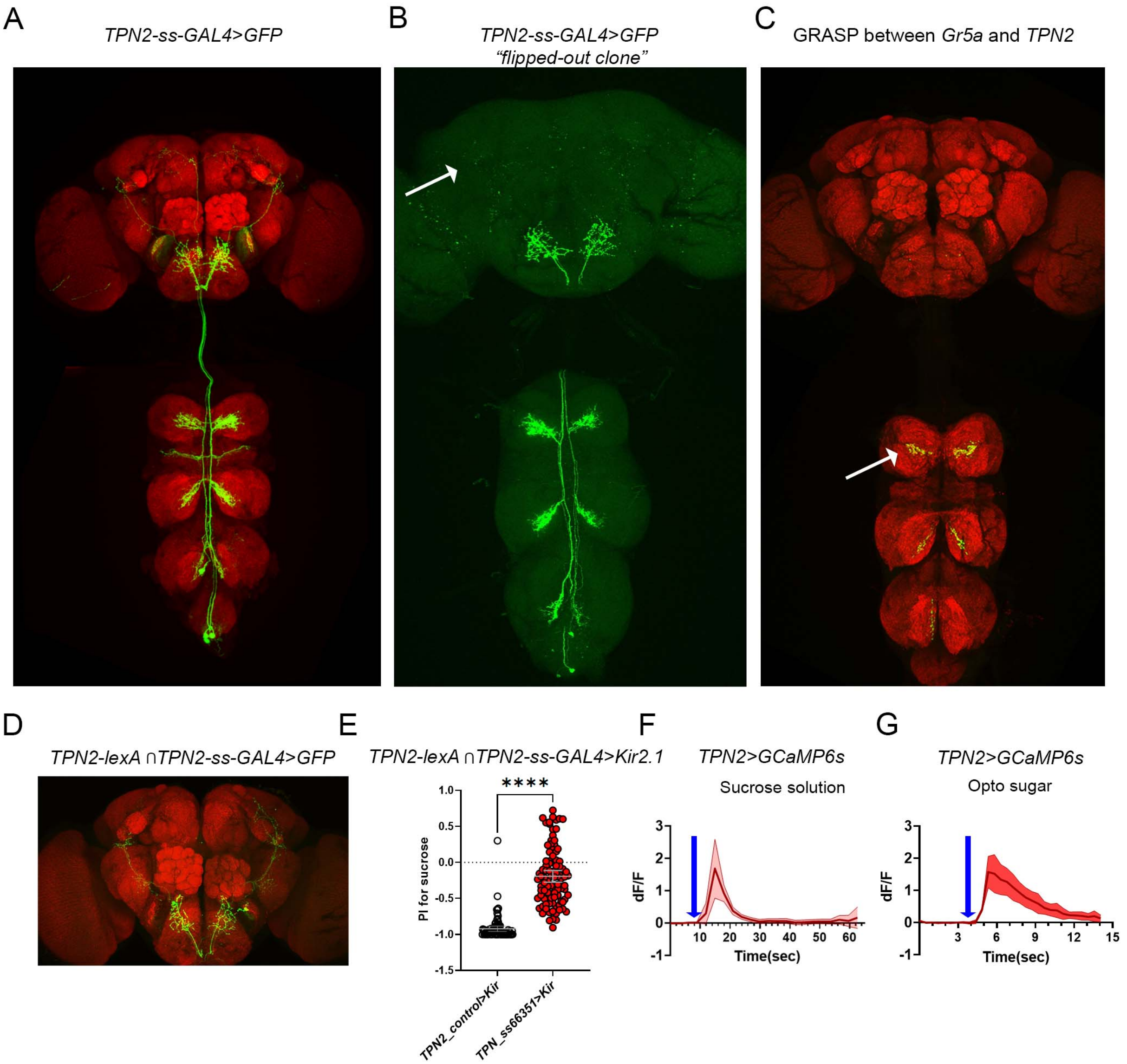

Figure S3. TPN2 neurons are post-synaptic target of leg sweet taste neurons.
(**A**) Confocal image of TPN2 neurons labeled by *ss66351*, showing projections to the superior lateral protocerebrum (SLP) and subesophageal zone (SEZ).
(**B**) Single TPN2 neuron generated by "random FLP-out", with its axon terminating in the SEZ but not the SLP (arrow).
(**C**) GRASP signals between TPN2 and *Gr5a*-expressing sweet neurons in T1–T3 ventral nerve cord segments (arrow).
(**D**) Intersectional labeling of *TPN2-LexA* with *ss66351*.
(**E**) PIs of control flies (n = 85) and flies with silenced intersected TPN2 neurons (n = 96).
(**F**) TPN2 responses to 150 mM sucrose (n = 8 flies; arrow indicates stimulus onset).
(**G**) TPN2 responses to optogenetic activation of *Gr64f*-expressing sweet neurons (n = 2 flies, with 2–3 technical repeats per fly).

Statistical tests in (**E)** were performed using unpaired two-sided Welch's t-tests. Significance is denoted as follows: ns: $p \geq 0.05$, *: $p < 0.05$, **: $p < 0.01$, ***: $p < 0.001$, and ****: $p < 0.0001$; all error bars represent 95% confidence intervals.

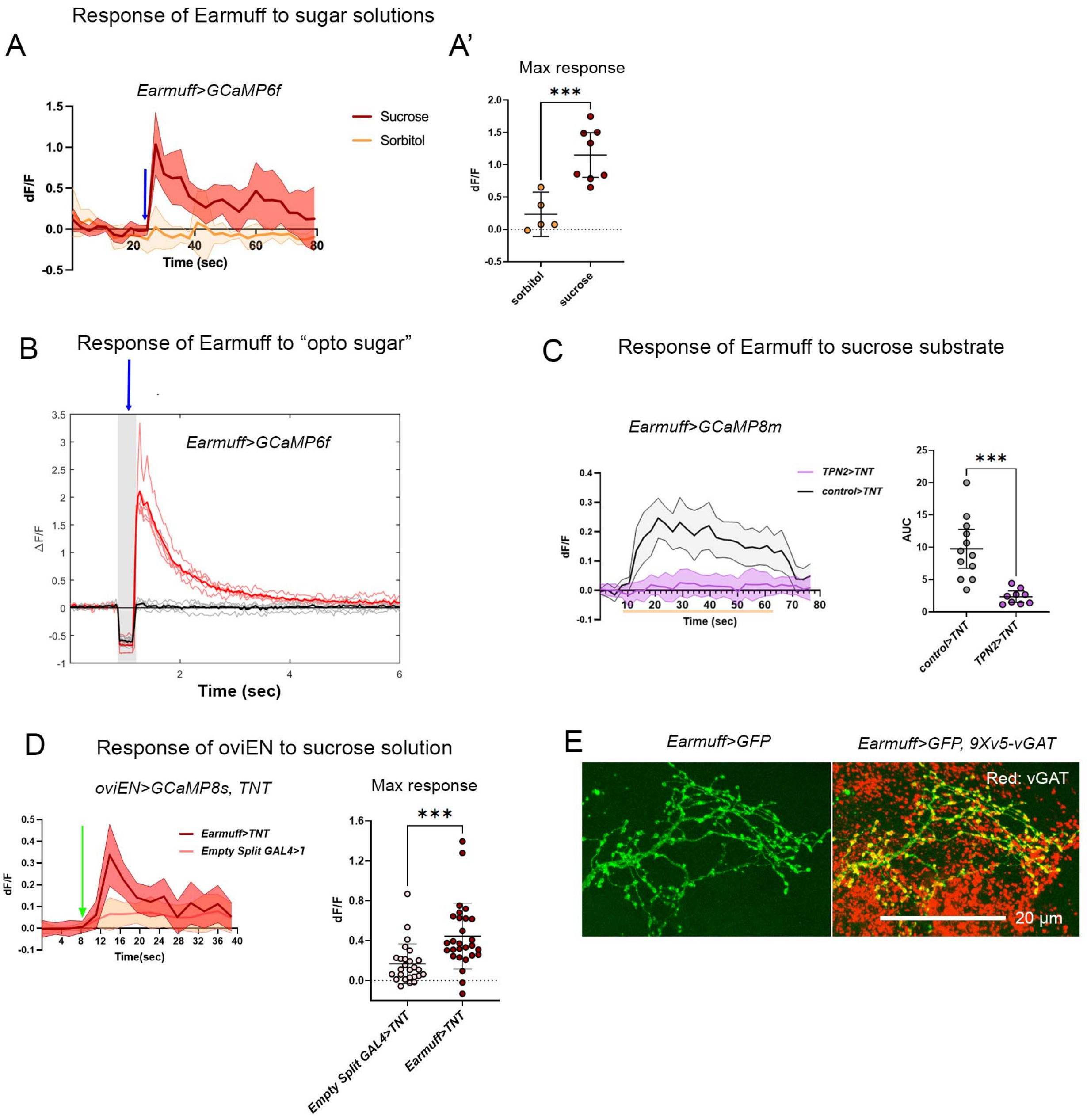

Figure S4. Earmuff sweet-taste responses require functional TPN2.

(**A** and **A′**) Traces of Earmuff responses to 150 mM sucrose and 150 mM sorbitol solutions (n = 8, 5) and corresponding peak ΔF/F; arrow indicates stimulus onset.

(**B**) Earmuff responses evoked by opto-activation of *Gr5a*-expressing neurons (n = 5); shaded region indicates five 10-ms red-light pulses at 50 Hz. PMT shutter was closed during stimulation.

(**C**) Earmuff responses to sucrose in controls (n = 12) and flies with TPN2 synaptic output blocked by tetanus toxin (n = 9). Blocking Earmuff itself (*control*>*TNT*) did not alter somatic Ca^{2+} signals.

(**D**) Sucrose-evoked responses of oviEN in flies expressing TNT in only oviEN versus in flies expressing TNT in both oviEN and Earmuff (n = 26, 28).

(**E**) Earmuff axonal processes (green) containing vesicular GABA transporter vGAT puncta (red).

Statistical tests in (**A**, **A′**, **C**, and **D**) were performed using unpaired two-sided Welch's t-tests. Significance is denoted as follows: ns: $p \geq 0.05$, *: $p < 0.05$, **: $p < 0.01$, ***: $p < 0.001$, and ****: $p < 0.0001$; all error bars represent 95% confidence intervals.

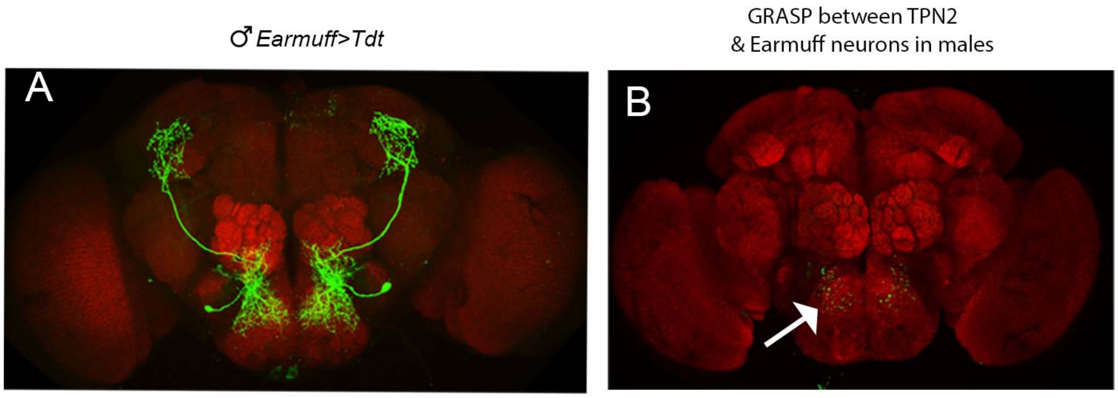

Figure S5. Anatomical Earmuff–TPN2 contacts in males.
(**A**) Confocal image of male Earmuff neuron morphology. Note that this image is reused from **Fig. 5M** as a reference.
(**B**) GRASP between TPN2 and Earmuff neurons in the male SEZ (green puncta and arrow).

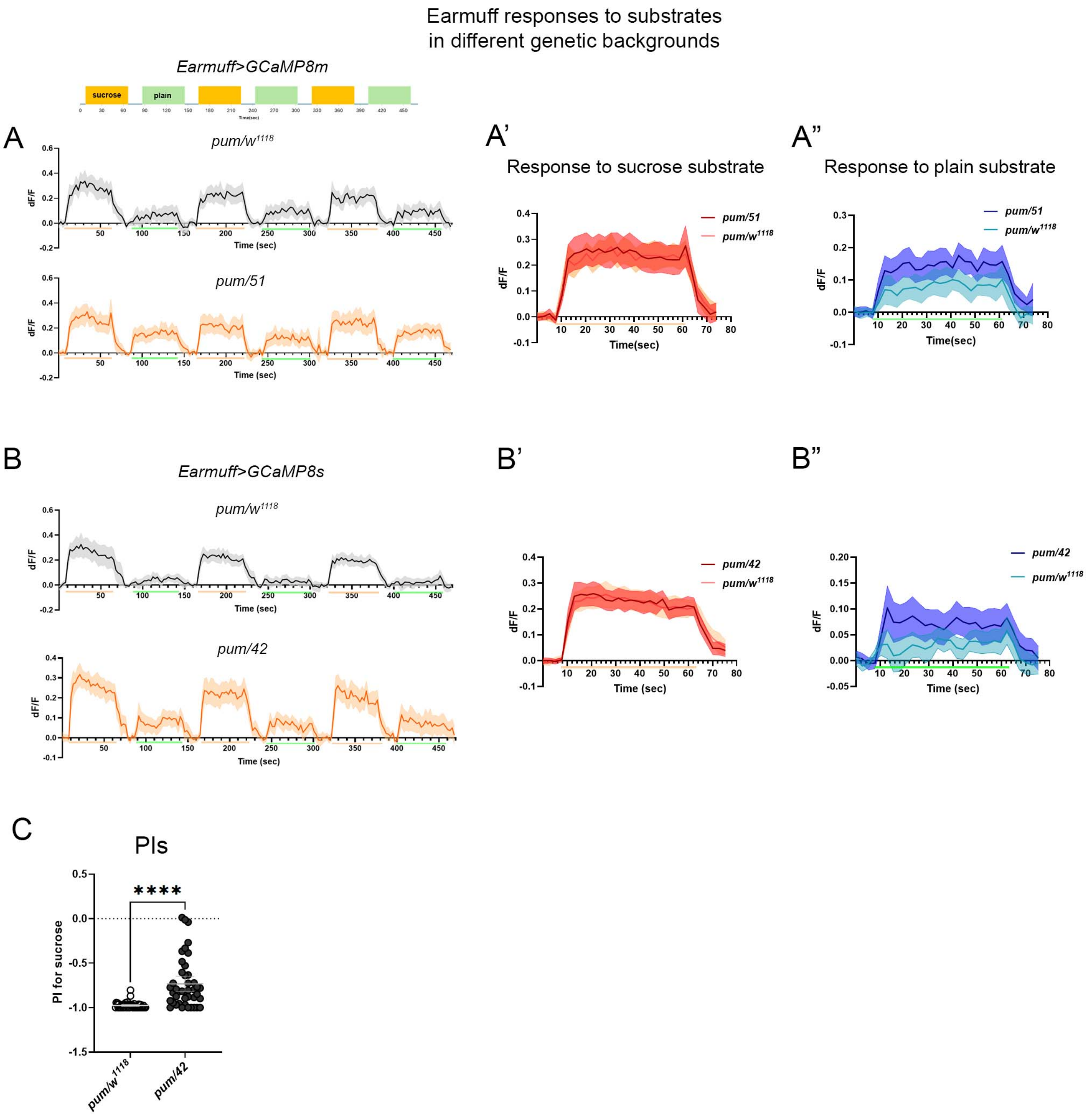

Figure S6. Earmuff responses in different wild-caught surrogates.
(**A** to **A″**) Traces and corresponding quantification from flies imaged in **Fig. 5B**.
(**B** to **B″**) Traces and corresponding quantification from flies imaged in **Fig. 5C**.
(**C**) PIs of control (*pum*/w^{1118}) and *pum/42* flies (n = 48, 43).

Statistical tests in (**C**) were performed using unpaired two-sided Welch's t-tests. Significance is denoted as follows: ns: $p \geq 0.05$, *: $p < 0.05$, **: $p < 0.01$, ***: $p < 0.001$, and ****: $p < 0.0001$; all error bars represent 95% confidence intervals.

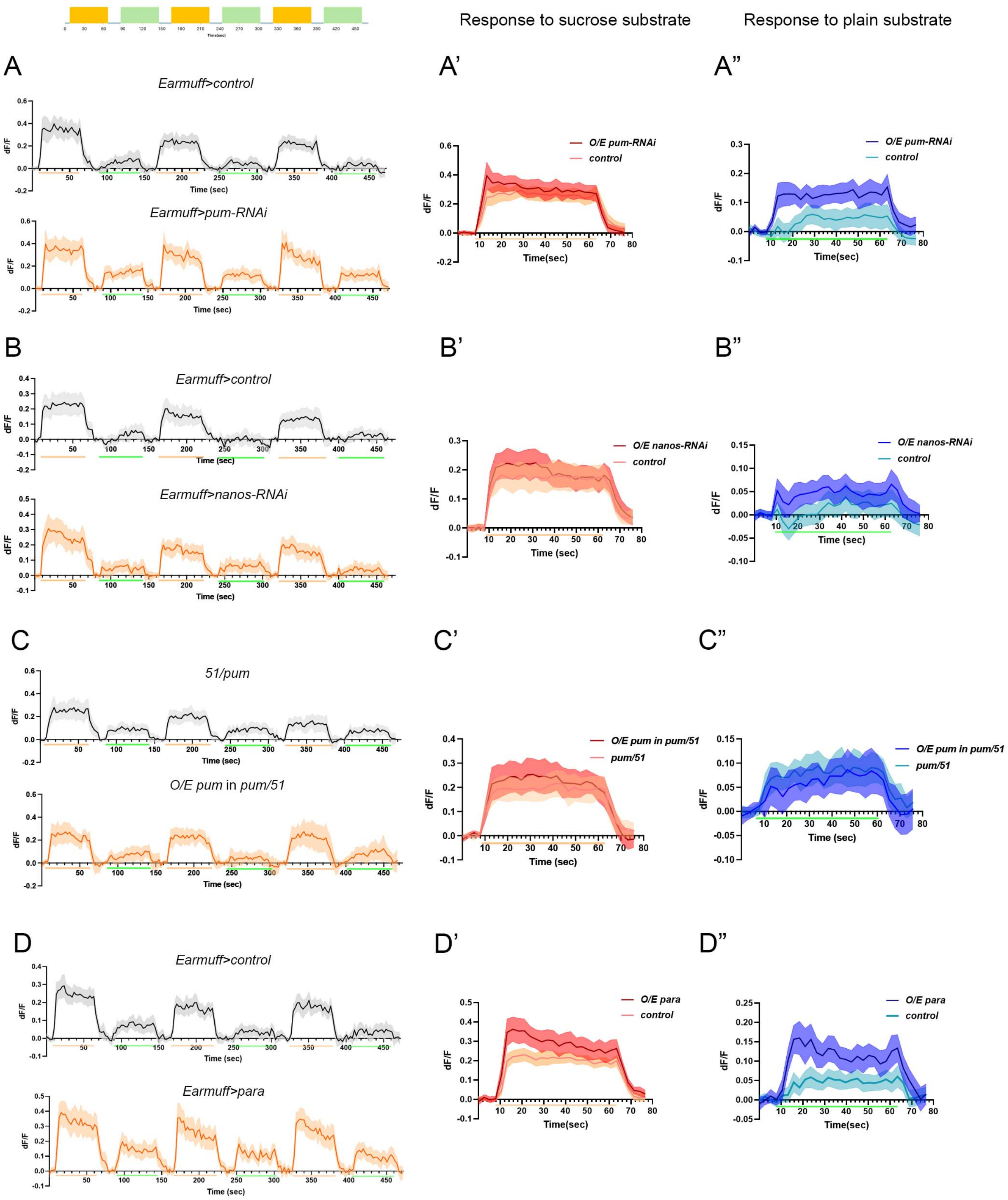

Figure S7. Earmuff responses under *pum*, *nos*, and *para* manipulations.

(**A** to **A″**) Traces and quantification from flies imaged for **Fig. 5E**.
(**B** to **B″**) Traces and quantification from flies imaged for **Fig. 5G**.
(**C** to **C″**) Traces and quantification from flies imaged for **Fig. 5I**.
(**D** to **D″**) Traces and quantification from flies imaged for **Fig. 5K**.

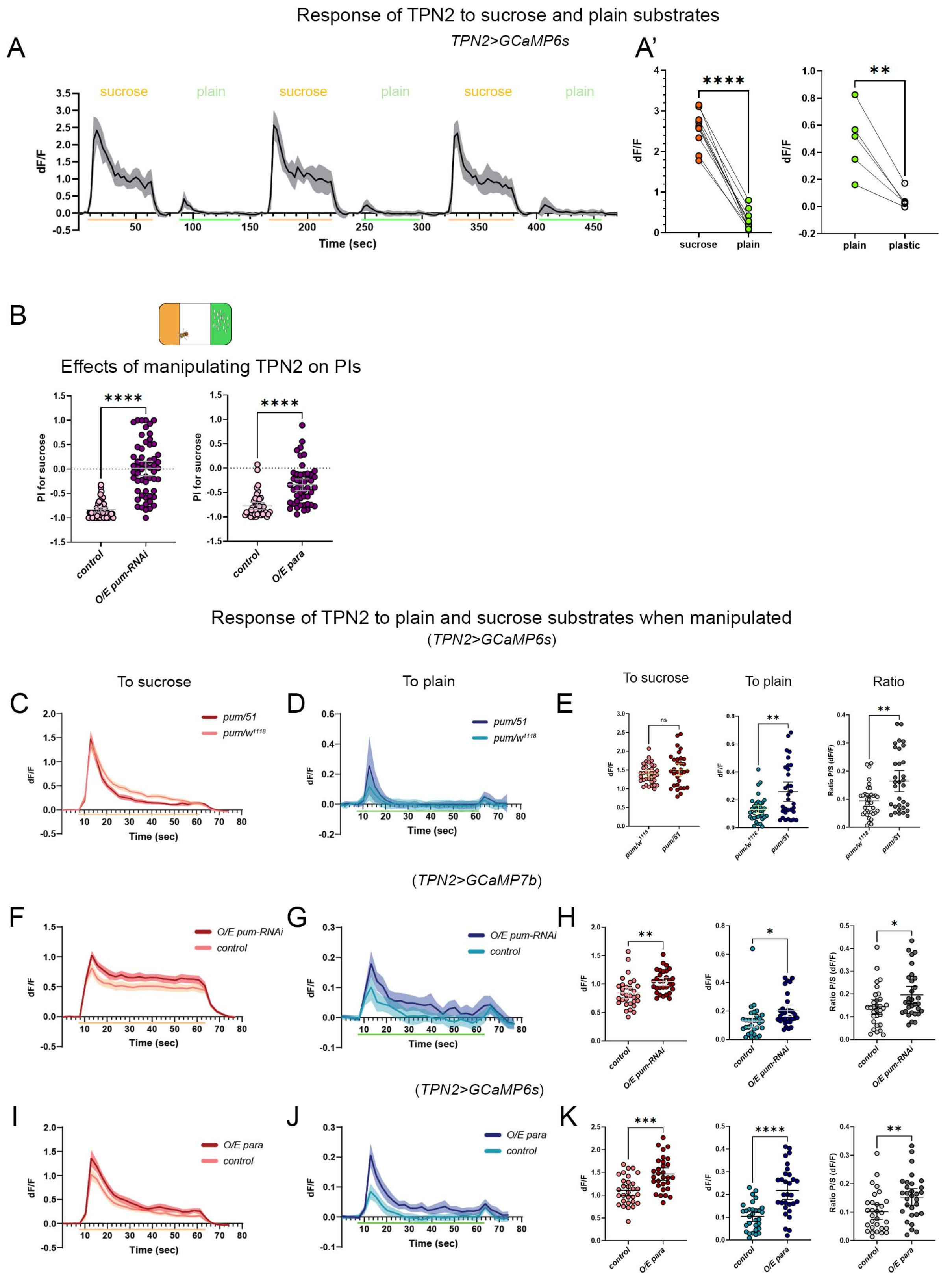

Figure S8. Substrate coding and behavioral impact of *pum* and *para* on TPN2.

(**A**) Traces of TPN2 responses to sucrose and plain substrates (n = 10).
(**A′**) Comparison of max TPN2 responses for sucrose versus plain (n = 10) and plain versus plastic (n = 5).
(**B**) PIs following TPN2-specific *pum* knockdown (n = 57) or *para* overexpression (n = 47) and corresponding controls (n = 54, 45).
(**C** to **E**) Traces of TPN2 responses in *pum*/w^{1118} (n = 36) and *pum/51* (n = 32), with quantification of max responses.
(**F** to **H**) Traces of TPN2 responses in control flies (n = 31) and *TPN2>pum-RNAi* flies (n = 31), with quantification of max responses.
(**I** to **K**) Traces of TPN2 responses in control flies (n = 30) and *TPN2>para* flies (n = 30), with quantification of max responses.

Statistical tests were performed using paired two-sided t-tests (**A′**) and unpaired two-sided Welch's t-tests (**B**, **E**, **H**, and **K**). Significance is denoted as follows: ns: p ≥ 0.05, *: p < 0.05, **: p < 0.01, ***: p < 0.001, and ****: p < 0.0001; all error bars represent 95% confidence intervals.

Variant		Genotype distribution by phenotype group		Beta	Potentially affected genes
Position	Seq. change	PI ≥ -0.1	PI ≤ -0.75		
3L:14590265	T->G	0/13/25	0/56/8	-0.47	HGTX (intron)
3L:15778514	C->A	0/13/32	0/61/17	-0.42	lncRNA:CR46197-CG6244 (intergenic_region)
3L:16013248	G->A	0/6/30	0/48/19	-0.44	lncRNA:CR43950 (downstream), lncRNA:CR45998 (upstream), mib1 (upstream), mib1-lncRNA:CR43950 (intergenic_region)
3L:16013253	GCA->G	0/6/36	0/48/25	-0.44	lncRNA:CR43950 (downstream), lncRNA:CR45998 (upstream), mib1 (upstream), mib1-lncRNA:CR43950 (intergenic_region)
3L:16047436	ATATG->A	0/11/34	0/57/17	-0.42	Diap1 (intron,upstream), Mbs (upstream), asRNA:CR45889 (downstream), lncRNA:CR43951 (upstream)
3L:18147732	A->T	0/14/33	1/59/15	-0.43	CG7330 (upstream), geko (downstream), geko-CG7330 (intergenic_region)
3L:18147735	C->A	0/14/33	1/59/15	-0.43	CG7330 (upstream), geko (downstream), geko-CG7330 (intergenic_region)
3L:25627853	A->AGGTGTTTAC...	0/13/32	0/61/16	-0.42	RR48317_transposable_element (upstream), lovit (intron)
3R:8107261	C->CA	0/15/28	0/61/10	-0.5	CG7900 (downstream), FBti0019335 (upstream), puc (intron)
3R:9079556	C->T	0/14/23	0/60/5	-0.52	pum (intron)
3R:11019925	*->ATGT	0/12/32	0/59/15	-0.42	side-VI (intron)
3R:11831160	TTC->T	1/16/30	0/64/11	-0.42	CG6959 (intron)
3R:12966855	T->C	0/14/32	0/64/15	-0.45	CG14384 (intron,upstream), CG7381 (downstream), Cyp304a1 (downstream), Spc25 (downstream), grsm (intron)
3R:13868303	*->T	4/11/28	30/28/14	-0.28	E5 (intron), lncRNA:CR46239 (upstream)
3R:14968498	T->G	0/14/31	0/60/13	-0.44	FBti0062141-lncRNA:CR45599 (intergenic_region)
3R:15125530	T->G	0/14/33	1/58/14	-0.45	CG42788 (intron)
3R:15783287	*->ACATGTG	4/11/22	31/27/9	-0.3	CG5614 (downstream), CG5614-CG9590 (intergenic_region), CG9590 (upstream)
3R:18634603	CAAAAAAAAA...->C	0/6/34	0/44/18	-0.46	CG14301 (intron), qin (intron)
3R:25532557	TC->*	3/12/21	31/25/12	-0.28	msi (intron)

Table S1. Variants significantly associated with preference index (PI).
The 19 variants listed are significantly associated with PI simultaneously under each of four adjusted p-value calculations (see Methods). The actual p-values are provided in **table S3**.

In auxiliary files:

Table S2. Key shared and 496 differentiating variants in the *pum* locus for lines 51 and 42.

Table S3. Longer list of 336 variants significantly associated with PI.
